# Transposable element polymorphisms improve prediction of complex agronomic traits in rice

**DOI:** 10.1007/s00122-022-04180-2

**Published:** 2022-08-05

**Authors:** Ioanna-Theoni Vourlaki, Raúl Castanera, Sebastián E. Ramos-Onsins, Josep M. Casacuberta, Miguel Pérez-Enciso

**Affiliations:** 1 Universitat Autònoma de Barcelona, Department of Animal Production, 08193 Bellaterra, Barcelona, Spain; 2 Centre for Research in Agricultural Genomics CSIC-IRTA-UAB-UB, 08193 Bellaterra, Barcelona, Spain; 3 Catalan Institute for Research and Advanced studies, ICREA, 08010 Barcelona, Spain

## Abstract

***Key message*:**

**Transposon insertion polymorphisms can improve prediction of complex agronomic traits in rice compared to using SNPs only, especially when accessions to be predicted are less related to the training set**.

**Abstract:**

Transposon insertion polymorphisms (TIPs) are significant sources of genetic variation. Previous work has shown that TIPs can improve detection of causative loci on agronomic traits in rice. Here, we quantify the fraction of variance explained by single nucleotide polymorphisms (SNPs) compared to TIPs, and we explore whether TIPs can improve prediction of traits when compared to using only SNPs. We used eleven traits of agronomic relevance from by five different rice population groups (Aus, Indica, Aromatic, Japonica, and Admixed), 738 accessions in total. We assess prediction by applying data split validation in two scenarios. In the within-population scenario, we predicted performance of improved Indica varieties using the rest of Indica accessions. In the across population scenario, we predicted all Aromatic and Admixed accessions using the rest of populations. In each scenario, Bayes C and a Bayesian reproducible kernel Hilbert space regression were compared. We find that TIPs can explain an important fraction of total genetic variance and that they also improve genomic prediction. In the across population prediction scenario, TIPs outperformed SNPs in nine out of the eleven traits analyzed. In some traits like leaf senescence or grain width, using TIPs increased predictive correlation by 30–50%. Our results evidence, for the first time, that TIPs genotyping can improve prediction on complex agronomic traits in rice, especially when accessions to be predicted are less related to training accessions.

**Supplementary Information:**

The online version contains supplementary material available at 10.1007/s00122-022-04180-2.

## Introduction

More than half of the world population consumes rice (*Oryza sativa*) in their daily diet. To secure nutritional requirements of a growing human population, the improvement of grain yield, both in quantity and in nutritional quality, is imperative. This is a significant challenge in the face of climate change and limited cultivable land. Current pace of rice genetic improvement may be too slow to meet these demands (Rosegrant and Cline 2003; Zhao et al. 2018). Genomic selection can be a useful tool to accelerate genetic progress (Meuwissen et al. 2001). Numerous studies in rice and in other plant species (Jighly et al. 2019; Tessema et al. 2020; Xu et al. 2020; Krishnappa et al. 2021) have already shown that genomic prediction (GP) can increase breeding speed. GP is particularly effective when traits are controlled by numerous loci which are difficult to map individually, such as yield and other traits of agronomic interest. For a recent review in rice, see Xu et al. (2021).

Conceptually, genomic prediction (GP) is a “large *p*, small *n*” scenario where the number of variables *p* (molecular markers) is typically far larger than the number of observations *n*. In this setting, either variables must be selected or restrictions on the solutions must be imposed, or a combination of both. Methods such as LASSO (Tibshirani 2011) or Bayes C (Habier et al. 2011) are examples of the first choice, whereas ridge regression or GBLUP (VanRaden 2008) involve restrictions on the square of solutions (L2 norm). Numerous metrics exist for measuring predictive ability. Among others, it can be measured as the correlation between predicted and observed phenotypes by splitting the data in training and test sets. Prediction accuracy is affected by different factors such as the size of the training data, heritability, similarity between training and testing populations, or choice of marker sets (Goddard and Hayes 2007; Robertsen et al. 2019; Xu et al. 2021).

In general, there is no consensus on which GP method is best. A recent review by Reinoso-Peláez et al. (2022) points at Reproducible Kernel Hilbert Space (RKHS) as the best overall method in plants. But there is variability. For instance, Tehseen et al. (2021) compared GBLUP, Ridge Regression (RR), LASSO, Elastic Net (EN), Bayesian Ridge Regression (BRR), Bayesian alphabet (A, B, C, …), RKHS for different traits, observing that no single method outperformed the rest for all traits. Kaler et al. (2022) conducted a comparative study among 11 different methods for two traits in soybean, rice, and maize, reporting better predictive abilities using Bayes B. Xu et al. (2018) found that GBLUP and LASSO performed best in hybrid breeding. Other authors have suggested integrating genomic prediction with crop growth models to evaluate the efficiency of phenotypic strategies and the impact of the different yield components on the prediction accuracy (Bustos-Korts et al. 2019; Cooper et al. 2016). Selecting SNPs based on genome-wide association studies (GWAS) has also been proposed, e.g., Spindel et al. (2016).

Irrespective of the algorithm chosen, single nucleotide polymorphisms (SNPs) are the main class of markers used so far in GP due to their genome-wide abundance and genotyping automatization. SNPs are not, however, the only source of phenotypic variability in the genome. In the last few years, data have accumulated on the importance of presence–absence variation and structural variation as a source of phenotypic variability in plants, including in rice (e.g., Fuentes et al. 2019). Transposon insertion polymorphisms (TIPs) can account for a major fraction of intraspecific structural variation, as it has been recently found in maize (Haberer et al. 2020). In fact, transposable elements are considered as one of the main drivers for plant genome variability, impacting on genome coding capacity and regulation in numerous ways (Lisch 2013). However, until the recent development and evaluation of reliable methods for calling TIPs from short-read resequencing data (Vendrell-Mir et al. 2019), it was not possible to use TIPs for GWAS approaches.

Importantly, recent studies in rice and in tomato have shown that the use of TIPs as genetic information can result in an increase of association signals as compared to SNPs in GWAS (Carpentier et al. 2019; Akakpo et al. 2020; Domínguez et al. 2020; Castanera et al. 2021). These results prompt us to investigate whether transposons can also improve prediction accuracy. For this purpose, we used the TIP genotypes from Castanera et al. (2021) and the phenotype database hosted in IRRI (Jackson 1997; Mansueto et al. 2017). Note that a better model fit, as observed in GWAS, does not necessarily imply a more accurate prediction and thus the question posed here is pertinent. Further, any improvement in prediction albeit small can translate into large genetic gains when accumulated through generations.

## Materials and methods

### Rice accessions and traits

The 738 accessions used in this study (Supplementary Table 1) are from the collection conserved at IRRI used for the 3000-rice genome project (Jackson 1997; Li et al. 2014) and were chosen because they were sequenced at least at 15 × depth. The 738 accessions retained pertain to all main rice population groups: Aus/Boro (AUS, *N* = 75), Indica (IND, *N* = 451), Japonica (JAP, *N* = 166), Aromatic (ARO, *N* = 17). The accessions that cannot be assigned to a specific rice group are categorized as Admixed (ADM, *N* = 29). We used the SNP-based group assignment from Sun et al. (2017) to identify the different subsets of this study.

Out of the 56 traits originally available at IRRI SNP-Seek database (https://snp-seek.irri.org/), we chose the 11 traits for which data were available in the 738 accessions selected. Some discrete traits were binned to balance the number of observations per class and time to flowering was log-transformed. Supplementary Table 2 shows basic statistics and transformations applied. Principal component analysis (PCA) for the 11 phenotypes was obtained with the “prcomp” function available in R.4.1.0 (Team 2021) environment. For plotting loading variables of PCA, package “factorextra” (Kassambara and Mundt 2020) and packages “ggrepel” (Slowikowski 2020) and “ggbiplot” (Vu 2011) for the biplot were used.

### Markers

A binary ped file format with the Core SNP dataset for all chromosomes was downloaded from the SNP-Seek database. The original dataset consisted of 404,388 bi-allelic SNPs from 3,034 rice accessions, including the 738 accessions selected. Markers with minor allele frequency $\le$ 0.01 and missing rate > 1% were filtered out using plink2 (Purcell et al. 2007; Chang et al. 2015). Missing genotypes were imputed using Beagle 5.2 with default parameters (Browning et al. 2018). The final dataset consisted of 228,871 SNPs, which were used for the analyses reported here. Of those, 50,485 SNPs were in gene regions.

Transposable elements (TEs) are divided in two main classes “copy and paste” (Class I TEs) or “cut and paste” Class II TEs. In rice, the most abundant Class I elements are RLX (LTR retrotransposons) and RIX (Non-LTR retrotransposons), whereas DTX (DNA TEs with terminal inverted repeats) and MITEs (Miniature Inverted-repeat Transposable Elements) are the most prevalent (Mao et al. 2000). Here we used markers from both classes, accounting for 94% of the TIPs described in Castanera et al. (2021). Class I TIPs were represented by 21,571 RLX and RIX markers. Class II consisted of 52,120 MITE and DTX markers. In contrast to SNPs, TIPs can only be genotyped as presence/absence, recoded consequently as 0/1, and defined as genomic windows with an average size of 1.2 kb. TIP windows were taken from Castanera et al. (2021) and are based on the intersection of the individual TE insertion regions predicted for each accession with genome-wide windows of a fixed size (1 kb, merging adjacent windows). These TIPs were further classified as genic or intergenic by intersecting the windows with MSU7 non-TE gene annotation (Kawahara et al. 2013). A TIP was considered genic if the window overlapped at least 1 bp with the gene feature. There were 17,034 genic MITE/DTX and 5,024 genic RLX/RIX TIPS. The remaining TIPs were considered intergenic.

MITEs amplify by bursts from individual elements creating highly homogeneous families, as previously reported in Arabidopsis (Santiago et al. 2002) and rice (Lu et al. 2017). Different bursts of amplification at different evolutionary times may have different prediction potential for particular phenotypes. In an attempt to study the potential predictive capacity of individual families, we created individual TIP genotype matrices for each of the 18 largest MITE families described in Castanera et al. (2021) (Supplementary Table 3). Each of these matrices included only TIPs originated from a single transposon, in this case MITE, family.

### Genetic variance inference

We fitted the following linear model in order to estimate genetic variance components explained by each marker set:

1a $${\bf{y}} = \mu + {\bf{Zu}}_1 + {\bf{Zu}}_2 + {\bf{Zu}}_3 + {\bf{e}}$$

where *μ* is the general mean, **y** is the phenotype vector of size *n*, the number of accessions, **Z** is an identity incidence matrix, **u**_**1**_, **u**_**2**_, **u**_**3**_ are random effects representing each of the marker groups, and **e** is the residual. We assume **u**_**1**_ ~ *N*(0, **K**_**S**_ $\sigma_S^2$), **u**_**2**_ ~ *N*(0, **K**_**M**_ $\sigma_M^2$), and **u**_**3**_ ~ *N*(0, **K**_**R**_ $\sigma_R^2$), where **K**_**S**_**, K**_**M**_**, K**_**R**_ are genomic relationship matrices obtained from SNPs, MITE/DTX, and RLX/RIX markers, respectively. These matrices were calculated using AGHMatrix (Amadeu et al. 2016). Model 1a was fitted with a Bayesian Reproducible Kernel Hilbert Space (RKHS, Herbrich et al. 1999) as implemented in BGLR package (Pérez and de Los Campos 2014) using default priors to estimate $\sigma_S^2$, $\sigma_M^2$, and $\sigma_R^2$.

### Genomic prediction

Plant breeding is primarily based on trials of new crosses, which can be lengthy and costly. The speed of development for new improved varieties depends largely on accuracy of prediction for new genotypes. We evaluated two distinct validation scenarios that cover two important issues: prediction of performance within population (rice group in this case) and prediction of individuals from different groups.

In the first scenario, we measured accuracy when predicting performance of improved Indica varieties (*N* = 48) using the rest of accessions, including non-improved Indica accessions. Accessions from IRRI core collection are classified as “improved,” “breeding and inbred lines,” and “traditional” varieties. We used this passport information to identify this subset of improved varieties. “Improved” Indica varieties correspond to most modern and commercial lines available at IRRI collection. In this scenario, performance to be predicted is from highly related accessions to those in the training set.

In the second scenario, we predicted performance of all Admixed (ADM, *N* = 29) and Aromatic (ARO, *N* = 17) accessions using the rest of groups. In this case, performance to be predicted is from accessions that may not be too related to accessions in the training set, and we expect prediction to be worse than in the former scenario. For instance, the ADM group is a small, highly heterogeneous collection of accessions.

The rationale for the first scenario is that new selected accessions can be crosses within the same population, and the breeder can be interested in designing new better performing crosses out of traditional varieties. The second scenario is more challenging, since we do not use any sample of the population to be predicted. These two scenarios, within and across populations, resemble main challenges faced in a breeding program. Note there are infinite designs for assessing predictive accuracy. For instance, we did not study prediction in Japonica because we preferred to focus on a larger number of traits, since genetic architecture is a main factor influencing predictive performance (Daetwyler et al. 2010).

Ample literature shows that no single method performs best for all traits and scenarios. Here, we compared two alternative modeling strategies: Bayesian RKHS as described above, and Bayes C. RKHS with the kernel employed here is equivalent to ridge regression and GBLUP, whereas Bayes C is a variable selection method. The two methods were applied to both predictive scenarios. For RKHS, we compared predictive performance using all markers (model 1a above) with submodels

1b $${\bf{y}} = \mu + {\bf{Zu}}_1 + {\bf{e}},$$

1c $${\bf{y}} = \mu + {\bf{Zu}}_2 + {\bf{e}},$$

and

1d $${\bf{y}} = \mu + {\bf{Zu}}_3 + {\bf{e}},$$

i.e., when using only SNPs (model 1b), only MITE/DTX (model 1c), or only RLX/RIX (model 1d) markers. For Bayes C, the complete model was:

2a $${\bf{y}} = \mu + {\bf{X}}_{\bf{S}} {{\beta }}_1 + {\bf{X}}_{\bf{M}} {{\beta }}_2 + {\bf{X}}_{\bf{R}} {{\beta }}_3 + {\bf{e}},$$

where **X**_**S**_, **X**_**M,**_ and **X**_**R**_ are the standardized genotypic values of each marker class; **β**_**1**_, **β**_**2,**_ and **β**_**3**_ are the corresponding vectors of effects for SNPs, MITE/DTX, and RLX/RIX markers, respectively. As with RKHS, three partial models were also evaluated:

2b $${\bf{y}} = \mu + {\bf{X}}_{\bf{S}} {\boldsymbol{\beta }}_1 + {\bf{e}},$$

2c $${\bf{y}} = \mu + {\bf{X}}_{\bf{M}} {\boldsymbol{\beta }}_2 + {\bf{e}},$$

and

2d $${\bf{y}} = \mu + {\bf{X}}_{\bf{R}} {\boldsymbol{\beta }}_3 + {\bf{e}}.$$

In Bayes C, a probability π of presence/absence of a SNP in the model is sampled from *π* ∼ Beta(*p*_0_, *π*_0_). Following Pérez and de Los Campos (2014, see their Tables 1 and S1), “the beta prior is parameterized in a way that the expected value by *E*(*π*) = *π*_0_; on the other hand, *p*_0_ can be interpreted as the number of prior counts (prior ‘successes’ plus prior ‘failures’).” Here we chose $p_0$ = 5 and *π*_0_ = 0.01.

**Table 1:** Means of posterior distributions of genetic variances explained by each marker set

Traits	All accessions (*N* = 738)			Indica accessions (*N* = 451)		
	$\sigma_S^2$	$\sigma_M^2$	$\sigma_R^2$	$\sigma_S^2$	$\sigma_M^2$	$\sigma_R^2$
Culm diameter	0.16	0.17*	0.16	0.13	0.17*	0.15
Culm strength	0.10	0.25*	0.16	0.11	0.19*	0.14
Flag leaf angle	0.22*	0.14	0.15	0.24*	0.14	0.14
Grain length	0.48*	0.11	0.11	0.41*	0.11	0.13
Grain width	0.49*	0.11	0.12	0.42*	0.11	0.14
Leaf length	0.26*	0.16	0.19	0.22*	0.16	0.19
Leaf senescence	0.12	0.25*	0.18	0.14	0.21*	0.16
Grain weight	0.40*	0.11	0.13	0.31*	0.12	0.13
Salt injury	0.10	0.11	0.12*	0.09	0.11*	0.11*
Time to flowering	0.45*	0.12	0.13	0.39*	0.13	0.13
Pan. threshability	0.11	0.13*	0.10	0.11	0.15*	0.11

$\sigma_S^2$: genetic variance explained by SNPs

$\sigma_M^2$: genetic variance explained by DNA transposon markers (MITE/DTX)

$\sigma_R^2$: genetic variance explained by retrotransposons (RLX/RIX)

Traits are scaled such that phenotypic variances are 1

*Best strategy

In a subset of cases, we evaluated whether using only genic SNPs improved prediction compared to using all available markers. Similarly, we conjectured that not all transposable elements are equally likely to cause phenotypic changes. We analyzed predictive performance of models containing TIPs from each of the largest 18 MITE families present in the rice genome (Supplementary Table 3). To avoid repetitive, lengthy results we make the additional analysis using two agronomic traits of high importance on rice breeding, time to flowering, and grain length. An earlier or later growing can determine seed production. Grain size-related traits such as grain length/width are important breeding targets since they affect the quality of the crop yield. These two traits may also represent alternative genetic architecture (Begum et al. 2015; Xu et al. 2015; Chen et al. 2021).

Using either RKHS or Bayes C, phenotypes to be predicted were removed from the dataset, the model fitted using the remaining phenotypes, and the correlation between predicted and observed phenotypes computed as a measure of predictive accuracy. From a practical point of view, it is important to assess whether predictions using TIPs or all markers are better than the state-of-the-art method, i.e., with SNPs only. To assess variability of results, we generated 10,000 bootstrap sampling with replacement from the corresponding pairs of phenotypes observed and predicted with each method and marker set. We then computed the correlation observed–predicted samples within each bootstrap sample and we counted how many times correlation using SNPs only was lower than with each alternative strategy. Phenotypic measurements and variables were centered and scaled to mean 0 and variance 1. BGLR was run for 100,000 iterations using default priors for RKHS. This number of iterations seemed enough to attain convergence (Supplementary Fig. 1).

## Results

### Descriptive analysis

**Fig. 1:**
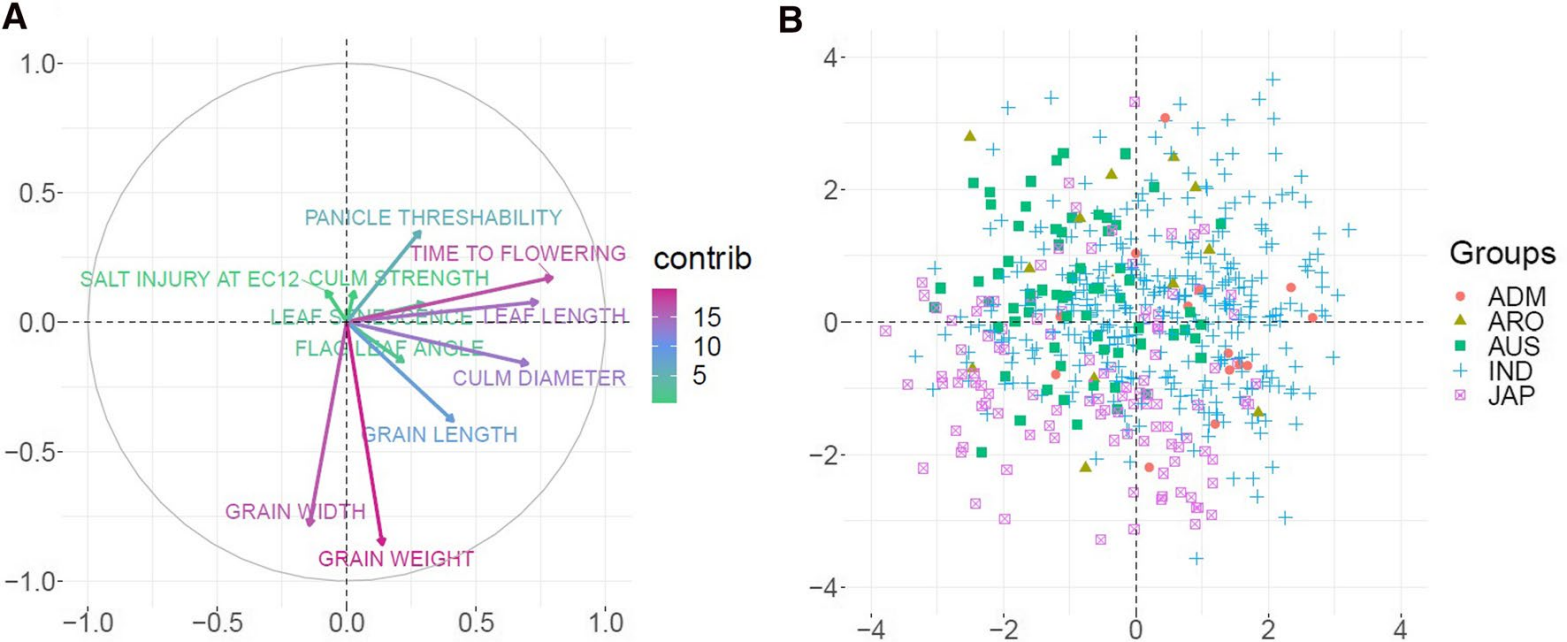
**a** PC loadings of each trait for the two first standardized principal components. **b** Plot showing the accessions projected. The first (*x*-axis) and second (*y*-axis) PCs explained 19% and 15.8% of variance, respectively

Figure 1a shows the loadings, i.e., the projections of variables into the lower-dimensional space, of each trait to the principal components. In the figure, the length of the arrow is proportional to trait contribution and the angle between arrows, to their correlation. An analysis in two principal components shows that the first component depends on grain width and grain weight, whereas culm diameter, time to flowering, and leaf length are the main contributors to the second component. The rest of traits contribute more modestly to total phenotypic variation. A sample projection (Fig. 1b) shows graphically how accessions differed in the traits studied. Supplementary Fig. 2 shows the differences in trait distributions across accessions. In general, populations differed for most traits although to varying extent. Figure 1b indicates, e.g., that Japonica accessions tend to have higher grain weights and widths, as they are projected in the lower part of the figure, and as shown in Supplementary Fig. 2.

### Genetic variance estimates

The genetic variance explained by each marker set measures its relative importance in determining the observed phenotypes. Here we prefer not to use the classical term “heritability” because a proper interpretation assumes panmixia, a condition not fulfilled here. Having these cautionary remarks in mind, Table 1 shows that transposons can explain a sizeable fraction of genetic variance, which was larger than that explained by SNPs in five out of 11 traits. In seven traits, $\sigma_S^2$ was smaller than the sum of $\sigma_M^2$ and $\sigma_R^2$. Results are presented when all accessions were analyzed together and when using only data from Indica, the largest group (*N* = 451). Note model (1a) assumes constant genetic variances across accessions, i.e., $\sigma_S^2$, $\sigma_M^2$, and $\sigma_R^2$ are the same in all rice groups. This is not necessarily the case. Nevertheless, variances were similar within Indica and across population groups.

### Genomic prediction

**Fig. 2:**
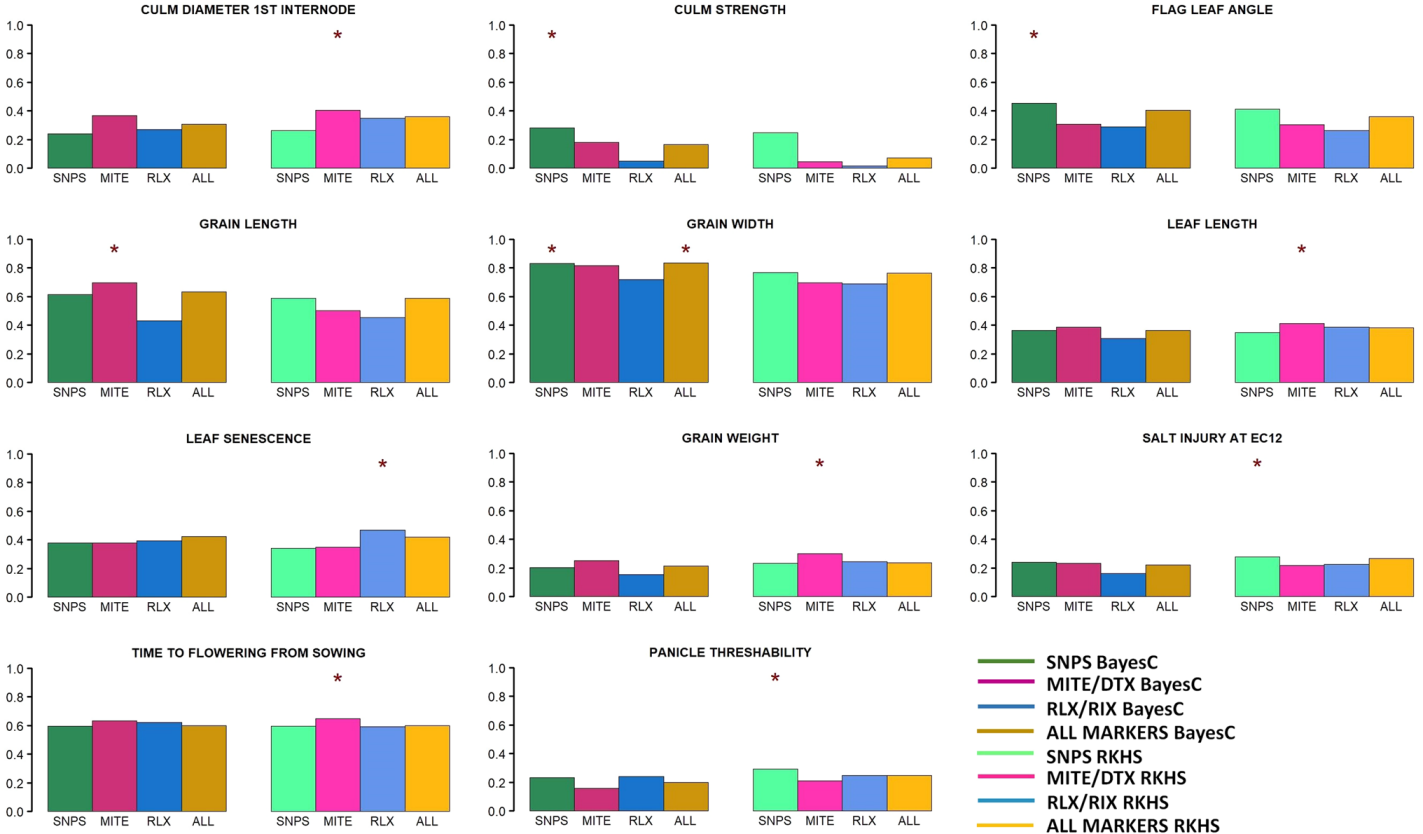
Correlation between observed and predicted phenotypes of Indica improved varieties. In each plot, the first four columns represent the correlation values using Bayes C, while the last four values correspond to RKHS method. Colors represent marker information utilized: green, SNPs; magenta, MITE/DTX; blue, RLX/RIX; brown, all markers. The asterisk shows the best option for each trait. (Color figure online)

We assess prediction in two validation scenarios that represent some of the main challenges in breeding, prediction within and across populations (see “Materials and methods”). In the first one, Indica improved varieties were predicted using the rest of accessions, including traditional Indica varieties. In this scenario, using TIPs increased prediction accuracy compared to using SNPs in six traits: culm diameter, grain length, leaf length, leaf senescence, grain weight, and time to flowering (Fig. 2).

**Fig. 3:**
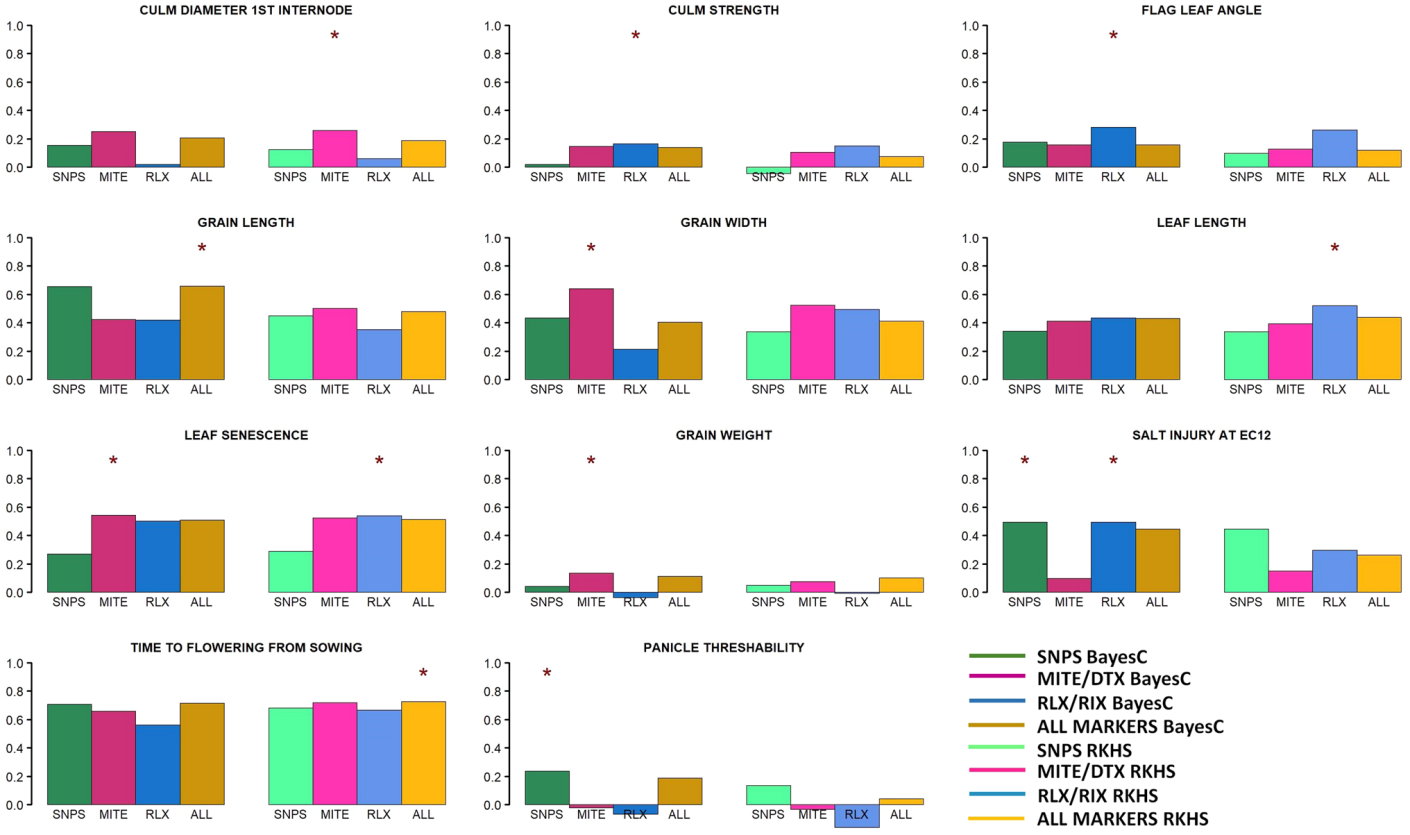
Correlation between observed and predicted phenotypes across accessions. All ADM and ADM accessions were predicted using the rest of groups. In each plot, the first four columns represent the correlation values using Bayes C, while the last four values correspond to RKHS method. Colors represent marker information utilized: green, SNPs; magenta, MITE/DTX; blue, RLX/RIX; brown, all markers. The asterisk shows the best option for each trait. (Color figure online)

In the second scenario, phenotypes of all ADM and ARO accessions were predicted given the rest of the accessions. TIPs were especially beneficial in this case: TIPs improved prediction upon using only SNPs in nine out of the 11 traits analyzed (Fig. 3). In some traits, such as grain width or leaf senescence, improvement in correlation using TIPs was remarkable, over 30%. In other traits, such as time to flowering, improvement was marginal. For some traits, notably grain weight and panicle threshability, prediction across populations was successful neither with SNPs nor with TIPs.

We computed the bootstrap probability that using TIPs, or all markers resulted in better predictions than using only SNPs (see “Materials and methods”). Results are given in Supplementary Tables 4 and 5 for the within and across population scenarios, respectively. Even if gains in accuracy shown in Figs. 2, 3 may seem small in some cases, results are consistent. For instance, increase in correlation for leaf length is ~ 15% when using MITE/DTX compared to SNPs in the within population scenario, a somewhat modest figure. But this result is confirmed in 80% of the bootstrap samples. In contrast, SNPs are far better than TIPs for culm strength and this is also confirmed in bootstrap samples (Supplementary Table 4, Fig. 2).

On average, prediction across populations was less accurate than within Indica in seven out of 11 traits and irrespective of marker set used (Figs. 2, 3). Importantly, gain using TIPs was larger in this scenario than in the within population scenario. Time to flowering and grain width were the traits for which prediction was most accurate. Nevertheless, prediction across populations for grain width was far less precise than within Indica. It is interesting to note that grain width and time to flowering are basically uncorrelated, but both contribute largely to total phenotypic variation (Fig. 1). This suggests that genomic prediction combined with transposable elements can be an effective tool for overall rice genetic improvement as it would enhance genetic progress in important agronomic traits.

Note that using all markers is not necessarily the best option for predictive purposes: it only outperformed the rest of approaches in three out of the 44 (= 11 traits × 2 methods × 2 predictive scenarios) analyses. This indicates that adding additional markers may contribute to overfitting and reduce model performance in prediction. Overfitting is a well-known phenomenon in the machine learning literature when the model is not properly regularized. This has been clearly observed with simulated data in a genomic prediction scenario (e.g., Pérez-Enciso et al. 2015).

Next, we wished to study how the different genetic architectures influence the statistical behavior of the three sets of markers. Bayes C is a variable selection method and so we reasoned that the number of markers entering the model and their effects would differ between traits. Broadly, estimates of marker effects were quite similar across traits (for the same type of marker) as can be seen in Supplementary Fig. 3. The only exception was grain width and grain length, where we observed much larger estimated effects for MITE/DTX and SNPs, respectively, in agreement with results in Fig. 3. In turn, there were larger differences between the probabilities (*d*) of entering the model for each marker type (Supplementary Fig. 4). This occurred despite setting equal priors for all types of markers (*p* = 0.01). This was not due only to the priors or different number of TIPs compared to SNPs, because the pattern differed between traits.

Using a subset of all markers available can improve prediction. For instance, the accuracy of a model which contains only the causative SNPs can approach one (Pérez-Enciso et al. 2015). The problem, of course, is that causative mutations cannot be identified in most cases. Several indirect approaches have been suggested instead. For instance, Spindel et al. (2016) proposed to perform prediction using the most associated markers, e.g., selected via a GWAS P-values. We did not evaluate this strategy here, although we did consider two alternative approaches for preselecting markers. In a first attempt, we examined whether using only gene-based markers improved prediction performance. To avoid multiplying analyses, we selected grain length and time to flowering. As can be seen in Table 2, gene-based markers outperform all markers in across population but minimally. The opposite was observed in the within population scenario.

**Table 2:** Predictive accuracy when using all or only gene-based markers

Prediction scenario	Trait^a^	Markers	Bayes C				RKHS			
			SNPS	MITE/DTX	RLX/RIX	ALL	SNPS	MITE/DTX	RLX/RIX	ALL
Across	GL	Genic	0.57	0.51	0.41	0.67*	0.47	0.54	0.26	0.48
		All	0.65	0.42	0.42	0.66	0.45	0.50	0.35	0.48
	TF	Genic	0.71	0.58	0.50	0.68	0.71	0.65	0.69	0.75*
		All	0.71	0.66	0.56	0.71	0.68	0.72	0.67	0.73
Within	GL	Genic	0.56	0.55	0.43	0.57	0.56	0.38	0.36	0.55
		All	0.61	0.69*	0.43	0.63	0.59	0.50	0.45	0.59
	TF	Genic	0.59	0.55	0.51	0.62	0.57	0.61	0.56	0.57
		All	0.59	0.63*	0.62	0.60	0.59	0.65	0.59	0.60

*Best strategy

^*a*^GL: grain length; TF: time to flowering

**Fig. 4:**
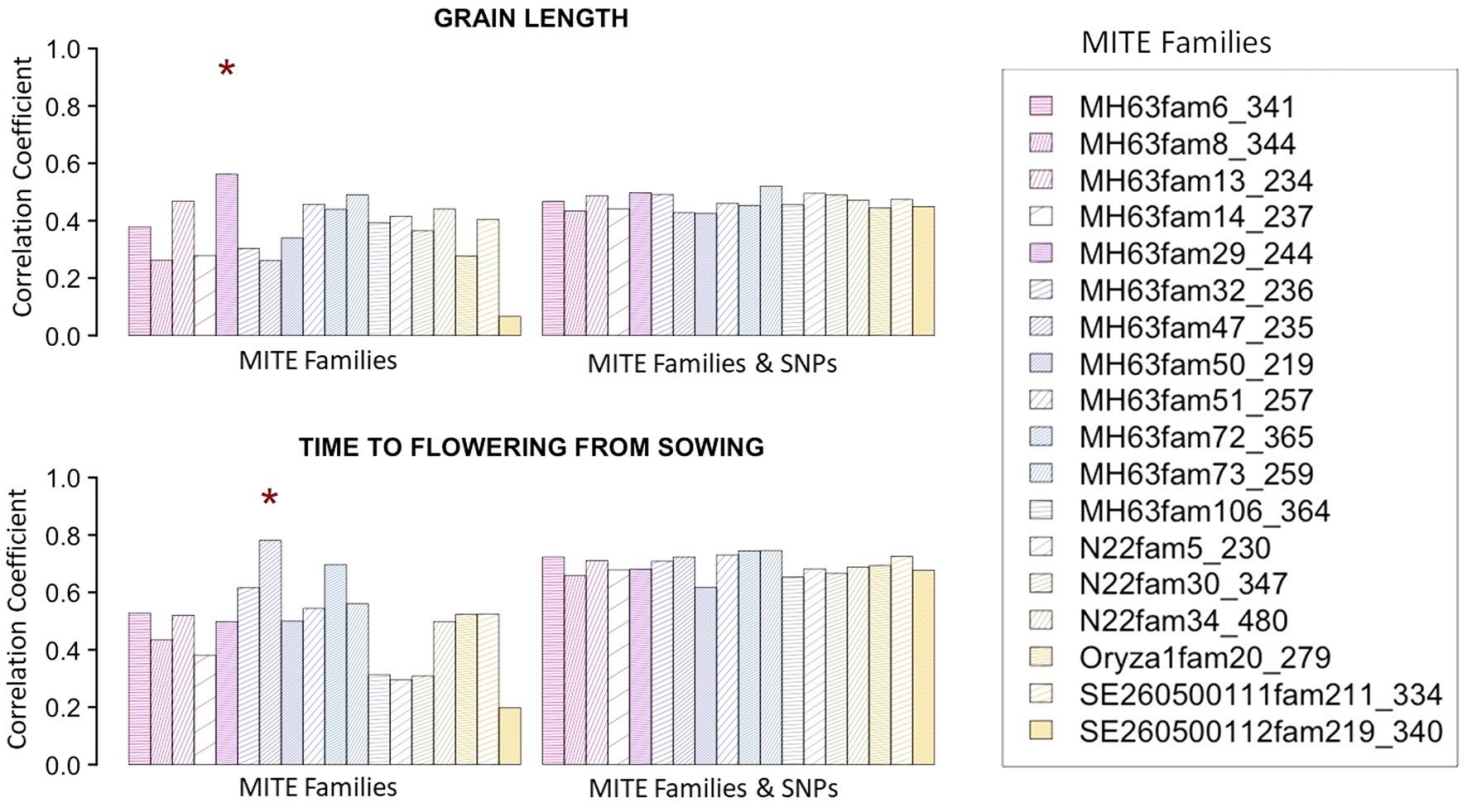
Predictive accuracy across populations using TIPS from each of 18 recognized MITE families. Each column corresponds to accuracy with one MITE family. Model included only MITEs or MITEs and all SNPs. The asterisk shows the best option

We also studied performance of TIPs pertaining to each of the 18 largest MITE families (Supplementary Table 3, see “Materials and methods”). Again, for brevity, we considered only prediction across accessions in grain length and time to flowering using RKHS (Fig. 4). The most relevant conclusion is that predictive performance can vary largely according to MITE family and that using SNPs on top of MITEs may not improve prediction. Prediction of time to flowering improved using MITE family MH63fam47_235 (MITE-adh type B-like superfamily) TIPs compared to using the full MITE/DTX set (Figs. 3, 4). Although it is tempting to conclude that a specific MITE family is enriched in genes affecting a given trait, one should be careful as disequilibrium can extend over long genome regions (Mather et al. 2007; Nachimuthu et al. 2015).

## Discussion

We have shown, for the first time to our knowledge, that transposable element polymorphisms can improve predictive accuracy for important agronomic traits in rice. The impact of using TIPs varied; here we found that they improved predictive performance in ~ 60% of the traits and scenarios considered. Table 3 presents a summary. The increase in accuracy also varied. Although the added benefit of using TIPs was sometimes modest, TIPs improved correlation by more than 30% in traits like grain width or leaf senescence.

**Table 3:** Maximum predictive accuracy and corresponding marker set

Trait	Scenario	
	Indica improved varieties	ARO/ADM accessions
Culm diameter	0.40 (MITE/DTX)	0.26 (MITE/DTX)
Culm strength	0.28 (SNPs)	0.16 (RLX/RIX)
Flag leaf angle	0.45 (SNPs)	0.28 (RLX/RIX)
Grain length	0.69 (MITE/DTX)	0.66 (ALL)
Grain width	0.83 (SNPs, ALL)	0.64 (MITE/DTX)
Leaf length	0.41 (MITE/DTX)	0.52 (RLX/RIX)
Leaf senescence	0.47 (RLX/RIX)	0.54 (MITE/DTX, RLX/RIX)
Grain weight	0.30 (MITE/DTX)	0.14 (MITE/DTX)
Salt injury	0.28 (SNPs)	0.49 (SNPs, MITE/DTX)
Time to flowering	0.65 (MITE/DTX)	0.73 (ALL)
Panicle threshability	0.29 (SNPs)	0.24 (SNPs)

All traits analyzed here have an economic impact in rice production. Unfortunately though, grain yield phenotypic data are not available for the 3 k rice panel, and how grain yield is affected by TIPs remains to be studied. This trait is largely affected by genotype × environment interaction, and so the relevance of TIPs may be harder to characterize. Among the traits studied, time to flowering is particularly important (Wang and Li 2019). Rice plants needs approximately, 3–6 months to grow, meaning that earlier or later growing can strongly affect the yield. Productivity is also determined by morphological trait such as grain weight (Chen et al. 2021). Grain weight in turn correlates with grain width, Fig. 1 (Li et al. 2021). Most of these traits are polygenic. Some traits like time to flowering, grain weight, grain width, and grain length seem controlled by large effect quantitative trait loci (Begum et al. 2015; Xu et al. 2015; Chen et al. 2021). For some traits, e.g., grain width, GP was quite accurate and we confirm that GP can largely enhance rice genetic progress, in agreement with previous results (Xu et al. 2021). For other traits, e.g., leaf length, GP accuracy was lower, although it is interesting to note that bootstrap sampling suggests that results are repeatable (Supplementary Tables 4, 5). Since plant breeding builds on cumulative progress over generations, even a small advantage can be highly relevant in the medium to long term.

The reasons behind the high capacity of TIPs to predict phenotypes, which in some cases is far better than SNPs, could be manifold. Transposable element insertions can have stronger effects than SNPs as some transposon types tend to localize near genes. Therefore, TIPs could be in some cases causative mutations linked to a specific trait. Indeed, transposable element insertions are known to have played a major role in plant genome evolution both in the wild and under breeding settings, and examples of TIP causative mutations for many agricultural important traits have been reported (Lisch 2013; Dubin et al. 2018). In some cases, the TIPs linked to the trait may be recent insertions and may not be in high LD with surrounding SNPs. This is what was shown in recent GWAS analyses performed with TIPs and SNPs in tomato and rice, where TIPs revealed associations with traits that are not detected with SNPs (Domínguez et al. 2020; Akakpo et al. 2020; Castanera et al. 2021). In contrast to SNP mutation rate, transposon activity is not constant over time, with bursts of transposition associated with stress situations or environmental stimuli (Dubin et al. 2018). Therefore, it can be hypothesized that the adaptation of a crop to a new environment, say as part of the breeding process, could be a period particularly prone to transposition activity (Baduel and Quadrana 2021). On the other hand, while SNPs accumulate relatively homogeneously throughout the genome, some TEs target gene-rich regions for integration, particularly RLXs and MITEs in rice (Castanera et al. 2021). Therefore, the potential for TEs to produce causal mutations and TIP associations with traits could be particularly high for some agronomic traits. Importantly, we found TIPs are especially helpful when prediction was across populations. These issues merit further research.

The main families of class I in rice are LTR-retrotransposons (RLX) and LINEs (RIX), whereas DNA transposons (DTX) and MITEs are the main components of rice class II TEs (Matsumoto et al. 2005). There are important structural and mechanistic differences between class I, or retrotransposons, and class II, or DNA transposons. Although both RLX and MITEs target genic regions for integration, their dynamics is very different. While RLXs have a high turnover and RLX TIPs are usually present at a very low frequency in the population, MITEs are maintained in the genome for longer evolutionary periods (Castanera et al. 2021). This suggests that although both types of TEs can be associated with traits in rice (Akakpo et al. 2020; Castanera et al. 2021), their capacity to predict phenotypes may differ. Certainly, our results show that MITE/DTX are more relevant than RLX/RIX for improving prediction (Table 3, Figs. 2, 3). It is finally interesting to note that a single MITE family of ~ 3 k TIPs can predict equally well a phenotype as well as 200 k SNPs (Fig. 4). In contrast, we did not find a consistent or large improvement in prediction when using only gene markers as compared to using all available polymorphisms, as reported also in humans (Visscher et al. 2021).

Some technical considerations should be borne in mind regarding our analyses. Ordinal traits (Supplementary Fig. 2) were treated as continuous. It has been known for decades that a threshold (logistic model) is theoretically a better choice for binary traits than standard linear models (Gianola and Foulley 1983). The logistic model is a class of the so-called generalized linear models, where the nonlinear relationship between parameters and observations becomes linear after applying a transformation, e.g., logit for binary traits. Despite their theoretical appeal, these models are more difficult to run than linear counterparts and may converge poorly. Empirical evidence generally shows small differences only (Matos et al. 1997; Olesen et al. 1994). Here, we observed (Supplementary Table 4) that a threshold model may have a small advantage over linear ones but not always. A second issue is the metrics to assess prediction. Here we chose correlation as it has a direct interpretation in terms of response to selection (Falconer and Mackay 1996) and has been widely used, but numerous other metrics exist. For instance, mean square error (RMSE) of prediction is also widely used. We computed RMSE (Supplementary Table 7, 8) and we found concordant results regarding the best marker set in 9 (within scenario) or 10 traits (across scenario) out of the 11 traits studied. These issues do not question our main, and most important conclusion regarding that TIPs can improve genomic prediction.

A prerequisite for the inclusion of TIPs in practical breeding programs is to automatize their genotyping. TIP genotyping should primarily target high-frequency TIPs in order to be as informative as possible, as it is usually done for SNPs as well. The application of TIP-Chip (Wheelan et al. 2006) or transposon insertion profiling (TIP-seq, Steranka et al. 2019), and TE-sequence capture (Quadrana et al. 2021) to hundreds or thousands of varieties should be cheap, as the sequencing coverage needed per sample is very small. Finally, given the dropping costs of genome sequencing, thousands of rice accessions are being re-sequenced and made public. TIPs could also be included in standard genotyping arrays (Wheelan et al. 2006) as a complement to SNPs. Given that TIPs from a single MITE family can be as efficient as 200 k SNPs in some traits (Fig. 4), perhaps only a small number of TIPs need to be included in the genotyping protocol.

In conclusion, we consistently observed that TIPs can increase predictive accuracy of agronomic traits in rice and do explain a non-negligible fraction of phenotypic variance. Notably, this improvement was larger when prediction was across populations than within Indica. Using markers positioned within genes did not seem to matter too much, although perhaps a more thorough analysis would be needed. In contrast, selecting TIPs from some transposon families did improve prediction. These are important results from a practical point of view and warrant developments to automatize TIP genotyping. From a biological point of view, new studies are needed to understand how TIPs affect complex trait variation. Improving predictive accuracy from molecular data is an important task since even small gains add up over generations and can make a big long-term difference. Assessing the importance of TIPs in other agronomic traits, such as grain yield across different environments, remains also to be studied. Once a plausible set of parameters linking TIPs, SNPs, and yield are estimated from real data, simulations can be used to optimize marker genotyping with SNPs and/or TIPs.

## Supplementary Information

Below is the link to the electronic supplementary material.

Supplementary file1 (DOCX 1409 kb)

## Data Availability

All data generated and software used during this study are included in a Github site https://github.com/ivourlaki/Transposable-element-polymorphisms-improve-prediction-of-complex-agronomic-traits-in-rice.git.
