# Supplementary material for: Transposable element polymorphisms improve prediction of complex agronomic traits in rice
Source: Theor Appl Genet. 2022 Aug 5;135(9):3211–22. doi: 10.1007/s00122-022-04180-2 (PMC9482605; doi:10.1007/s00122-022-04180-2)
Supplement: Supplementary file 1 — Supplementary file1 (DOCX 1409 kb) [file 122_2022_4180_MOESM1_ESM.docx]

Supplementary Information

# **Transposable element polymorphisms improve prediction of**

# **complex agronomic traits in rice**

**Supplementary Table 1** Accessions used in this study.

| Accession | Group | Status* | Country | Accession | Group | Status* | Country |
| --- | --- | --- | --- | --- | --- | --- | --- |
| IRIS 313-10000 | IND | I | South_Korea | IRIS 313-11684 | IND | T | Thailand |
| IRIS 313-10010 | IND | T | Fiji | IRIS 313-11685 | IND | T | Thailand |
| IRIS 313-10020 | AUS | T | Sri_Lanka | IRIS 313-11686 | IND | T | Thailand |
| IRIS 313-10026 | IND | NA | Madagascar | IRIS 313-11687 | IND | T | Thailand |
| IRIS 313-10059 | JAP | NA | South_Korea | IRIS 313-11689 | JAP | NA | South_Korea |
| IRIS 313-10078 | JAP | T | Japan | IRIS 313-11691 | JAP | T | Bhutan |
| IRIS 313-10097 | JAP | T | South_Korea | IRIS 313-11692 | IND | T | Taiwan |
| IRIS 313-10109 | IND | T | Nigeria | IRIS 313-11693 | IND | T | Taiwan |
| IRIS 313-10114 | IND | I | Burundi | IRIS 313-11700 | IND | T | Thailand |
| IRIS 313-10134 | IND | NA | Thailand | IRIS 313-11704 | IND | T | Thailand |
| IRIS 313-10158 | IND | NA | Ecuador | IRIS 313-11705 | IND | T | Thailand |
| IRIS 313-10171 | IND | NA | China | IRIS 313-11706 | IND | T | Thailand |
| IRIS 313-10177 | IND | NA | China | IRIS 313-11707 | IND | T | Thailand |
| IRIS 313-10179 | IND | NA | China | IRIS 313-11708 | IND | T | Thailand |
| IRIS 313-10189 | IND | NA | China | IRIS 313-11709 | IND | T | Thailand |
| IRIS 313-10221 | IND | NA | China | IRIS 313-11710 | IND | T | Thailand |
| IRIS 313-10228 | JAP | NA | China | IRIS 313-11711 | IND | T | Thailand |
| IRIS 313-10235 | IND | I | Philippines | IRIS 313-11712 | AUS | NA | India |
| IRIS 313-10237 | IND | I | Philippines | IRIS 313-11716 | IND | T | Guinea |
| IRIS 313-10260 | IND | NA | Paraguay | IRIS 313-11717 | IND | T | Indonesia |
| IRIS 313-10301 | IND | I | Brazil | IRIS 313-11719 | IND | NA | Thailand |
| IRIS 313-10327 | JAP | NA | Peru | IRIS 313-11720 | IND | NA | Thailand |
| IRIS 313-10332 | IND | I | Indonesia | IRIS 313-11721 | IND | NA | Thailand |
| IRIS 313-10333 | IND | I | Indonesia | IRIS 313-11722 | IND | T | Bangladesh |
| IRIS 313-10337 | IND | I | Indonesia | IRIS 313-11723 | IND | T | Guinea |
| IRIS 313-10352 | IND | I | Colombia | IRIS 313-11724 | IND | T | Guinea |
| IRIS 313-10392 | IND | I | Philippines | IRIS 313-11725 | JAP | I | Japan |
| IRIS 313-10394 | IND | I | Philippines | IRIS 313-11727 | IND | NA | China |
| IRIS 313-10397 | IND | I | Colombia | IRIS 313-11728 | IND | NA | China |
| IRIS 313-10423 | IND | NA | Myanmar | IRIS 313-11730 | IND | NA | China |
| IRIS 313-10440 | JAP | NA | Philippines | IRIS 313-11731 | IND | NA | China |
| IRIS 313-10458 | IND | P | China | IRIS 313-11732 | IND | NA | China |
| IRIS 313-10469 | JAP | I | Japan | IRIS 313-11733 | IND | NA | China |
| IRIS 313-10477 | IND | NA | China | IRIS 313-11734 | IND | NA | China |
| IRIS 313-10503 | IND | NA | China | IRIS 313-11736 | JAP | T | Philippines |
| IRIS 313-10509 | IND | NA | Africa | IRIS 313-11737 | AUS | T | India |
| IRIS 313-10511 | IND | T | Philippines | IRIS 313-11738 | IND | T | India |
| IRIS 313-10515 | IND | I | Taiwan | IRIS 313-11739 | JAP | T | Ghana |
| IRIS 313-10518 | IND | NA | Myanmar | IRIS 313-11740 | IND | I | Ghana |
| IRIS 313-10519 | IND | P | India | IRIS 313-11741 | IND | T | Sri_Lanka |
| IRIS 313-10524 | IND | NA | na | IRIS 313-11742 | AUS | NA | India |
| IRIS 313-10526 | IND | NA | India | IRIS 313-11744 | IND | NA | China |
| IRIS 313-10534 | AUS | P | India | IRIS 313-11745 | IND | NA | China |
| IRIS 313-10541 | JAP | P | Guinea-Bissau | IRIS 313-11746 | IND | NA | China |
| IRIS 313-10542 | IND | NA | India | IRIS 313-11747 | IND | NA | China |
| IRIS 313-10544 | IND | NA | India | IRIS 313-11748 | IND | NA | China |
| IRIS 313-10547 | IND | P | Myanmar | IRIS 313-11750 | IND | NA | China |
| IRIS 313-10550 | IND | i | Indonesia | IRIS 313-11751 | IND | NA | China |
| IRIS 313-10560 | IND | NA | China | IRIS 313-11752 | IND | NA | China |
| IRIS 313-10561 | IND | NA | China | IRIS 313-11753 | IND | NA | China |
| IRIS 313-10563 | JAP | P | na | IRIS 313-11754 | ADM | NA | Madagascar |
| IRIS 313-10564 | JAP | P | Japan | IRIS 313-11755 | JAP | NA | Liberia |
| IRIS 313-10570 | JAP | NA | Japan | IRIS 313-11756 | JAP | NA | Madagascar |
| IRIS 313-10576 | IND | NA | Sierra_Leone | IRIS 313-11757 | IND | NA | Madagascar |
| IRIS 313-10577 | JAP | T | Philippines | IRIS 313-11758 | IND | NA | Ivory_Coast |
| IRIS 313-10578 | JAP | T | Philippines | IRIS 313-11759 | JAP | NA | Ivory_Coast |
| IRIS 313-10582 | JAP | T | Philippines | IRIS 313-11760 | ADM | NA | Madagascar |
| IRIS 313-10602 | AUS | P | Bangladesh | IRIS 313-11761 | ADM | NA | Ivory_Coast |
| IRIS 313-10603 | AUS | P | Bangladesh | IRIS 313-11762 | IND | NA | Madagascar |
| IRIS 313-10605 | AUS | P | Bangladesh | IRIS 313-11763 | IND | I | Cameroon |
| IRIS 313-10609 | IND | T | Sri_Lanka | IRIS 313-11764 | IND | I | Liberia |
| IRIS 313-10614 | IND | NA | Hong_Kong | IRIS 313-11767 | ADM | NA | Madagascar |
| IRIS 313-10617 | JAP | NA | na | IRIS 313-11772 | IND | T | Madagascar |
| IRIS 313-10623 | AUS | NA | Nepal | IRIS 313-11773 | IND | NA | Gambia |
| IRIS 313-10628 | IND | T | India | IRIS 313-11784 | IND | T | Sierra_Leone |
| IRIS 313-10642 | JAP | P | Japan | IRIS 313-11786 | IND | T | Gambia |
| IRIS 313-10652 | IND | T | Laos | IRIS 313-11787 | IND | T | Gambia |
| IRIS 313-10654 | IND | P | Laos | IRIS 313-11788 | JAP | T | Philippines |
| IRIS 313-10657 | JAP | NA | Laos | IRIS 313-11789 | ADM | T | Madagascar |
| IRIS 313-10664 | IND | NA | India | IRIS 313-11790 | JAP | T | Madagascar |
| IRIS 313-10666 | IND | NA | India | IRIS 313-11791 | IND | T | Madagascar |
| IRIS 313-10671 | AUS | NA | India | IRIS 313-11792 | JAP | T | Madagascar |
| IRIS 313-10675 | AUS | T | India | IRIS 313-11794 | ADM | NA | Madagascar |
| IRIS 313-10677 | JAP | P | Japan | IRIS 313-11795 | IND | NA | China |
| IRIS 313-10682 | IND | T | Laos | IRIS 313-11796 | IND | NA | China |
| IRIS 313-10687 | IND | T | Malaysia | IRIS 313-11797 | IND | NA | China |
| IRIS 313-10688 | IND | T | Malaysia | IRIS 313-11798 | IND | NA | China |
| IRIS 313-10693 | JAP | T | Indonesia | IRIS 313-11799 | IND | NA | China |
| IRIS 313-10697 | IND | T | Malaysia | IRIS 313-11800 | JAP | NA | China |
| IRIS 313-10703 | JAP | T | Malaysia | IRIS 313-11801 | IND | NA | China |
| IRIS 313-10706 | IND | T | Malaysia | IRIS 313-11802 | IND | NA | China |
| IRIS 313-10707 | IND | T | Malaysia | IRIS 313-11804 | IND | NA | China |
| IRIS 313-10710 | JAP | T | Surinam | IRIS 313-11805 | IND | NA | China |
| IRIS 313-10712 | JAP | NA | Ivory_Coast | IRIS 313-11806 | IND | NA | China |
| IRIS 313-10718 | AUS | T | Sri_Lanka | IRIS 313-11807 | IND | I | Colombia |
| IRIS 313-10723 | IND | NA | Senegal | IRIS 313-11809 | AUS | T | Kenya |
| IRIS 313-10725 | IND | NA | Senegal | IRIS 313-11810 | IND | T | Kenya |
| IRIS 313-10726 | IND | NA | Senegal | IRIS 313-11811 | ADM | T | Kenya |
| IRIS 313-10727 | IND | NA | Senegal | IRIS 313-11812 | IND | T | Kenya |
| IRIS 313-10728 | IND | NA | Senegal | IRIS 313-11813 | IND | T | Kenya |
| IRIS 313-10733 | IND | NA | Nepal | IRIS 313-11814 | IND | T | Kenya |
| IRIS 313-10744 | JAP | T | Indonesia | IRIS 313-11815 | IND | T | Kenya |
| IRIS 313-10748 | IND | T | Vietnam | IRIS 313-11816 | IND | T | Myanmar |
| IRIS 313-10756 | IND | I | India | IRIS 313-11817 | IND | T | Myanmar |
| IRIS 313-10762 | IND | T | Indonesia | IRIS 313-11819 | IND | T | Myanmar |
| IRIS 313-10771 | ADM | T | Indonesia | IRIS 313-11820 | IND | T | Myanmar |
| IRIS 313-10774 | IND | T | Indonesia | IRIS 313-11821 | IND | T | India |
| IRIS 313-10778 | IND | T | Indonesia | IRIS 313-11822 | IND | T | India |
| IRIS 313-10779 | IND | T | Indonesia | IRIS 313-11823 | IND | T | India |
| IRIS 313-10786 | ADM | T | Indonesia | IRIS 313-11824 | IND | T | India |
| IRIS 313-10787 | ADM | T | Indonesia | IRIS 313-11825 | ARO | T | India |
| IRIS 313-10789 | JAP | T | Indonesia | IRIS 313-11829 | JAP | T | Pakistan |
| IRIS 313-10793 | JAP | T | Indonesia | IRIS 313-11832 | JAP | T | Thailand |
| IRIS 313-10794 | JAP | T | Indonesia | IRIS 313-11833 | IND | T | Thailand |
| IRIS 313-10797 | IND | T | Indonesia | IRIS 313-11835 | IND | T | Thailand |
| IRIS 313-10798 | JAP | T | Indonesia | IRIS 313-11836 | IND | T | Thailand |
| IRIS 313-10802 | JAP | T | Indonesia | IRIS 313-11840 | IND | T | Thailand |
| IRIS 313-10805 | JAP | T | Indonesia | IRIS 313-11842 | IND | T | Thailand |
| IRIS 313-10806 | IND | T | Indonesia | IRIS 313-11848 | IND | T | Malaysia |
| IRIS 313-10810 | IND | T | Indonesia | IRIS 313-11854 | IND | NA | China |
| IRIS 313-10813 | IND | T | Indonesia | IRIS 313-11866 | IND | NA | China |
| IRIS 313-10814 | IND | T | Indonesia | IRIS 313-11867 | IND | NA | China |
| IRIS 313-10816 | JAP | T | Indonesia | IRIS 313-11870 | IND | NA | China |
| IRIS 313-10820 | IND | T | Indonesia | IRIS 313-11877 | IND | NA | China |
| IRIS 313-10822 | IND | T | Indonesia | IRIS 313-11878 | IND | NA | China |
| IRIS 313-10824 | IND | T | Indonesia | IRIS 313-11882 | IND | NA | China |
| IRIS 313-10825 | IND | NA | NA | IRIS 313-11887 | IND | I | Philippines |
| IRIS 313-10827 | JAP | T | Philippines | IRIS 313-11897 | JAP | T | Thailand |
| IRIS 313-10834 | JAP | I | India | IRIS 313-11900 | JAP | NA | Thailand |
| IRIS 313-10835 | IND | I | India | IRIS 313-11902 | IND | NA | Indonesia |
| IRIS 313-10840 | JAP | T | South_Korea | IRIS 313-11909 | IND | NA | China |
| IRIS 313-10845 | AUS | NA | India | IRIS 313-11916 | IND | T | Sri_Lanka |
| IRIS 313-10847 | IND | NA | India | IRIS 313-11919 | IND | T | India |
| IRIS 313-10850 | ADM | NA | India | IRIS 313-11920 | IND | NA | Thailand |
| IRIS 313-10851 | ARO | NA | India | IRIS 313-11924 | JAP | NA | Thailand |
| IRIS 313-10852 | AUS | NA | India | IRIS 313-11927 | IND | NA | Thailand |
| IRIS 313-10857 | AUS | NA | India | IRIS 313-11929 | JAP | T | Philippines |
| IRIS 313-10858 | AUS | NA | India | IRIS 313-11930 | ADM | P | Nigeria |
| IRIS 313-10859 | AUS | NA | India | IRIS 313-11935 | IND | T | Cambodia |
| IRIS 313-10861 | AUS | NA | India | IRIS 313-11939 | IND | T | Burkina_Faso |
| IRIS 313-10863 | IND | NA | India | IRIS 313-11940 | IND | T | Burkina_Faso |
| IRIS 313-10870 | JAP | NA | India | IRIS 313-11941 | IND | T | Burkina_Faso |
| IRIS 313-10871 | AUS | NA | India | IRIS 313-11945 | IND | T | Bangladesh |
| IRIS 313-10872 | ADM | NA | India | IRIS 313-11949 | IND | NA | China |
| IRIS 313-10883 | ARO | NA | India | IRIS 313-11950 | IND | NA | China |
| IRIS 313-10888 | JAP | NA | India | IRIS 313-11953 | IND | NA | China |
| IRIS 313-10889 | JAP | NA | India | IRIS 313-11955 | IND | NA | China |
| IRIS 313-10891 | AUS | NA | India | IRIS 313-11959 | IND | T | Philippines |
| IRIS 313-10892 | AUS | NA | India | IRIS 313-11962 | IND | NA | Thailand |
| IRIS 313-10894 | AUS | NA | India | IRIS 313-11966 | IND | T | China |
| IRIS 313-10895 | JAP | NA | India | IRIS 313-11978 | IND | I | Philippines |
| IRIS 313-10900 | IND | T | Cambodia | IRIS 313-11979 | IND | I | Philippines |
| IRIS 313-10912 | IND | T | Cambodia | IRIS 313-11988 | IND | T | Sierra_Leone |
| IRIS 313-10916 | JAP | T | Cambodia | IRIS 313-11989 | IND | T | Brunei_Darussalam |
| IRIS 313-10918 | ADM | T | Philippines | IRIS 313-11994 | JAP | T | Philippines |
| IRIS 313-10921 | IND | NA | Laos | IRIS 313-11999 | IND | T | Cambodia |
| IRIS 313-10922 | JAP | NA | Laos | IRIS 313-12000 | IND | T | Cambodia |
| IRIS 313-10923 | JAP | T | Thailand | IRIS 313-12010 | IND | NA | China |
| IRIS 313-10927 | AUS | T | Nepal | IRIS 313-12024 | IND | NA | na |
| IRIS 313-10928 | IND | T | Thailand | IRIS 313-12033 | IND | NA | China |
| IRIS 313-10930 | AUS | P | Bangladesh | IRIS 313-12040 | IND | T | Cambodia |
| IRIS 313-10936 | JAP | NA | Indonesia | IRIS 313-12041 | IND | T | Cambodia |
| IRIS 313-10937 | IND | T | Indonesia | IRIS 313-12044 | IND | T | Cambodia |
| IRIS 313-10938 | IND | T | Indonesia | IRIS 313-12045 | JAP | T | Indonesia |
| IRIS 313-10941 | IND | NA | Indonesia | IRIS 313-12048 | IND | T | Indonesia |
| IRIS 313-10942 | IND | T | Indonesia | IRIS 313-12052 | IND | T | India |
| IRIS 313-10944 | IND | T | Indonesia | IRIS 313-12054 | JAP | I | China |
| IRIS 313-10948 | ADM | NA | Indonesia | IRIS 313-12058 | IND | T | Cambodia |
| IRIS 313-10949 | JAP | NA | Indonesia | IRIS 313-12060 | JAP | NA | China |
| IRIS 313-10954 | IND | T | Indonesia | IRIS 313-12061 | JAP | NA | China |
| IRIS 313-10960 | JAP | T | Indonesia | IRIS 313-12068 | JAP | T | Indonesia |
| IRIS 313-10961 | IND | T | Indonesia | IRIS 313-12071 | JAP | NA | Laos |
| IRIS 313-10965 | AUS | T | Bangladesh | IRIS 313-12094 | ARO | T | Bangladesh |
| IRIS 313-10968 | IND | NA | Brazil | IRIS 313-12097 | IND | T | Cambodia |
| IRIS 313-10975 | IND | T | Bangladesh | IRIS 313-12101 | IND | T | Cambodia |
| IRIS 313-10980 | IND | T | Bangladesh | IRIS 313-12108 | JAP | NA | Malaysia |
| IRIS 313-10984 | IND | T | Bangladesh | IRIS 313-12118 | ADM | NA | Madagascar |
| IRIS 313-10986 | IND | T | Bangladesh | IRIS 313-12121 | IND | T | Laos |
| IRIS 313-10988 | IND | P | India | IRIS 313-12127 | IND | T | Laos |
| IRIS 313-10990 | IND | T | Philippines | IRIS 313-12128 | IND | T | Laos |
| IRIS 313-10994 | JAP | T | Philippines | IRIS 313-12129 | JAP | T | Laos |
| IRIS 313-10995 | IND | T | Indonesia | IRIS 313-12131 | IND | NA | Laos |
| IRIS 313-10997 | IND | T | Indonesia | IRIS 313-12134 | JAP | T | Laos |
| IRIS 313-10999 | JAP | T | Indonesia | IRIS 313-12135 | IND | NA | Malaysia |
| IRIS 313-11005 | JAP | T | Indonesia | IRIS 313-12141 | AUS | T | Bangladesh |
| IRIS 313-11007 | JAP | T | Indonesia | IRIS 313-12146 | IND | T | Cambodia |
| IRIS 313-11016 | AUS | T | Bangladesh | IRIS 313-12164 | JAP | T | Cambodia |
| IRIS 313-11024 | AUS | T | Pakistan | IRIS 313-12183 | AUS | T | Nepal |
| IRIS 313-11027 | AUS | T | Pakistan | IRIS 313-12188 | IND | NA | Laos |
| IRIS 313-11032 | ARO | T | Pakistan | IRIS 313-12190 | IND | T | Laos |
| IRIS 313-11033 | IND | NA | Pakistan | IRIS 313-12193 | IND | T | Laos |
| IRIS 313-11034 | AUS | NA | Pakistan | IRIS 313-12228 | JAP | T | Laos |
| IRIS 313-11035 | AUS | NA | Pakistan | IRIS 313-12234 | IND | NA | China |
| IRIS 313-11037 | AUS | T | Pakistan | IRIS 313-12258 | JAP | T | Laos |
| IRIS 313-11038 | IND | P | China | IRIS 313-12259 | IND | NA | Laos |
| IRIS 313-11039 | IND | I | China | IRIS 313-12262 | JAP | NA | Laos |
| IRIS 313-11040 | IND | NA | India | IRIS 313-12268 | IND | T | Myanmar |
| IRIS 313-11042 | IND | NA | India | IRIS 313-12275 | IND | NA | China |
| IRIS 313-11043 | IND | NA | Malaysia | IRIS 313-12281 | JAP | NA | Madagascar |
| IRIS 313-11044 | JAP | NA | Malaysia | IRIS 313-12287 | IND | T | Myanmar |
| IRIS 313-11045 | JAP | NA | Malaysia | IRIS 313-12289 | JAP | T | Myanmar |
| IRIS 313-11046 | JAP | NA | Malaysia | IRIS 313-12291 | IND | T | Myanmar |
| IRIS 313-11047 | AUS | P | Bangladesh | IRIS 313-12300 | IND | T | Laos |
| IRIS 313-11048 | AUS | P | Bangladesh | IRIS 313-12303 | IND | T | Laos |
| IRIS 313-11049 | AUS | P | Bangladesh | IRIS 313-12305 | IND | T | Laos |
| IRIS 313-11050 | AUS | P | Bangladesh | IRIS 313-12307 | JAP | NA | Laos |
| IRIS 313-11051 | AUS | P | Bangladesh | IRIS 313-12312 | JAP | NA | Laos |
| IRIS 313-11052 | AUS | P | Bangladesh | IRIS 313-12321 | JAP | T | Laos |
| IRIS 313-11053 | AUS | P | Bangladesh | IRIS 313-12323 | JAP | T | Laos |
| IRIS 313-11054 | AUS | P | Bangladesh | IRIS 313-12334 | IND | T | Laos |
| IRIS 313-11055 | AUS | P | Bangladesh | IRIS 313-12349 | JAP | NA | Laos |
| IRIS 313-11056 | AUS | P | Bangladesh | IRIS 313-12350 | JAP | T | Laos |
| IRIS 313-11057 | AUS | P | Bangladesh | IRIS 313-12351 | JAP | T | Laos |
| IRIS 313-11058 | AUS | P | Bangladesh | IRIS 313-12354 | IND | T | Laos |
| IRIS 313-11059 | AUS | P | Bangladesh | IRIS 313-12355 | IND | T | Laos |
| IRIS 313-11062 | ARO | P | Bangladesh | IRIS 313-15900 | IND | I | Philippines |
| IRIS 313-11063 | AUS | P | Bangladesh | IRIS 313-15908 | AUS | I | Colombia |
| IRIS 313-11064 | AUS | P | Bangladesh | IRIS 313-15910 | JAP | I | United_States |
| IRIS 313-11066 | ARO | P | Bangladesh | IRIS 313-7638 | IND | NA | Madagascar |
| IRIS 313-11079 | IND | T | Laos | IRIS 313-7646 | ADM | NA | Madagascar |
| IRIS 313-11081 | IND | T | Laos | IRIS 313-7650 | IND | NA | Madagascar |
| IRIS 313-11083 | IND | T | Laos | IRIS 313-7685 | IND | I | Philippines |
| IRIS 313-11085 | IND | NA | Laos | IRIS 313-7688 | IND | I | Philippines |
| IRIS 313-11089 | IND | NA | Cambodia | IRIS 313-7689 | IND | I | Philippines |
| IRIS 313-11094 | JAP | T | Laos | IRIS 313-7719 | IND | NA | Mali |
| IRIS 313-11095 | IND | T | Laos | IRIS 313-7722 | ADM | NA | Madagascar |
| IRIS 313-11097 | IND | T | Philippines | IRIS 313-7725 | ADM | T | Madagascar |
| IRIS 313-11098 | IND | NA | Sierra_Leone | IRIS 313-7795 | ADM | T | Madagascar |
| IRIS 313-11102 | JAP | NA | Liberia | IRIS 313-7797 | IND | I | Philippines |
| IRIS 313-11103 | JAP | NA | Liberia | IRIS 313-7799 | IND | NA | Madagascar |
| IRIS 313-11104 | JAP | NA | Liberia | IRIS 313-7808 | IND | I | Senegal |
| IRIS 313-11112 | AUS | T | Bangladesh | IRIS 313-7816 | IND | I | Senegal |
| IRIS 313-11113 | IND | T | Bangladesh | IRIS 313-7850 | JAP | NA | Madagascar |
| IRIS 313-11118 | IND | T | Vietnam | IRIS 313-7866 | ADM | I | Colombia |
| IRIS 313-11129 | IND | NA | Myanmar | IRIS 313-7876 | JAP | T | Philippines |
| IRIS 313-11151 | IND | I | Myanmar | IRIS 313-7883 | JAP | T | Indonesia |
| IRIS 313-11160 | IND | NA | Liberia | IRIS 313-7902 | JAP | I | Philippines |
| IRIS 313-11189 | ARO | NA | Soviet_Union | IRIS 313-7909 | ADM | I | Philippines |
| IRIS 313-11191 | AUS | T | Sri_Lanka | IRIS 313-7911 | IND | I | Philippines |
| IRIS 313-11194 | IND | NA | Thailand | IRIS 313-7914 | JAP | I | Ivory_Coast |
| IRIS 313-11202 | JAP | I | China | IRIS 313-7924 | ADM | I | Bolivia |
| IRIS 313-11205 | IND | T | Bangladesh | IRIS 313-7933 | ADM | T | Nepal |
| IRIS 313-11221 | IND | P | Bangladesh | IRIS 313-7994 | JAP | T | Madagascar |
| IRIS 313-11224 | IND | P | Bangladesh | IRIS 313-8010 | JAP | I | Philippines |
| IRIS 313-11226 | IND | P | Bangladesh | IRIS 313-8024 | JAP | NA | Italy |
| IRIS 313-11228 | IND | P | Bangladesh | IRIS 313-8037 | JAP | NA | Italy |
| IRIS 313-11229 | IND | P | Bangladesh | IRIS 313-8064 | JAP | I | Argentina |
| IRIS 313-11231 | IND | P | Bangladesh | IRIS 313-8066 | JAP | NA | Italy |
| IRIS 313-11234 | IND | T | Philippines | IRIS 313-8074 | JAP | I | Australia |
| IRIS 313-11238 | JAP | NA | Brazil | IRIS 313-8085 | JAP | NA | Spain |
| IRIS 313-11239 | IND | I | Indonesia | IRIS 313-8115 | JAP | NA | Portugal |
| IRIS 313-11240 | IND | I | India | IRIS 313-8118 | JAP | NA | Portugal |
| IRIS 313-11241 | IND | I | Bangladesh | IRIS 313-8119 | JAP | NA | Bulgaria |
| IRIS 313-11242 | IND | I | India | IRIS 313-8123 | JAP | NA | Portugal |
| IRIS 313-11244 | IND | NA | India | IRIS 313-8125 | JAP | NA | Bulgaria |
| IRIS 313-11245 | IND | P | India | IRIS 313-8127 | JAP | NA | Bulgaria |
| IRIS 313-11247 | IND | I | India | IRIS 313-8129 | JAP | NA | Bulgaria |
| IRIS 313-11249 | IND | I | Philippines | IRIS 313-8140 | JAP | NA | China |
| IRIS 313-11251 | IND | I | Philippines | IRIS 313-8151 | JAP | P | Portugal |
| IRIS 313-11252 | IND | NA | India | IRIS 313-8166 | JAP | NA | France |
| IRIS 313-11253 | IND | I | Surinam | IRIS 313-8167 | JAP | I | France |
| IRIS 313-11256 | IND | NA | India | IRIS 313-8168 | JAP | NA | France |
| IRIS 313-11257 | ADM | NA | India | IRIS 313-8172 | ADM | NA | Philippines |
| IRIS 313-11258 | ARO | NA | India | IRIS 313-8173 | JAP | I | United_States |
| IRIS 313-11260 | IND | NA | India | IRIS 313-8177 | JAP | NA | Italy |
| IRIS 313-11262 | IND | NA | India | IRIS 313-8185 | JAP | NA | Italy |
| IRIS 313-11263 | ADM | NA | India | IRIS 313-8204 | JAP | I | United_States |
| IRIS 313-11264 | IND | NA | India | IRIS 313-8208 | JAP | NA | Portugal |
| IRIS 313-11265 | AUS | NA | India | IRIS 313-8293 | IND | NA | Senegal |
| IRIS 313-11266 | IND | NA | India | IRIS 313-8305 | IND | T | India |
| IRIS 313-11267 | IND | NA | India | IRIS 313-8312 | IND | T | Malaysia |
| IRIS 313-11269 | IND | NA | India | IRIS 313-8321 | AUS | P | Bangladesh |
| IRIS 313-11270 | ARO | NA | India | IRIS 313-8323 | JAP | I | United_States |
| IRIS 313-11271 | IND | NA | India | IRIS 313-8326 | ARO | NA | India |
| IRIS 313-11273 | IND | NA | India | IRIS 313-8332 | IND | NA | India |
| IRIS 313-11274 | AUS | NA | India | IRIS 313-8341 | IND | NA | Vietnam |
| IRIS 313-11275 | IND | NA | India | IRIS 313-8349 | IND | T | Bangladesh |
| IRIS 313-11277 | AUS | NA | India | IRIS 313-8356 | JAP | T | Philippines |
| IRIS 313-11278 | IND | NA | India | IRIS 313-8381 | JAP | T | Malaysia |
| IRIS 313-11279 | IND | NA | India | IRIS 313-8386 | IND | NA | India |
| IRIS 313-11280 | IND | NA | India | IRIS 313-8391 | IND | NA | Burkina_Faso |
| IRIS 313-11281 | IND | NA | India | IRIS 313-8407 | IND | T | Malaysia |
| IRIS 313-11285 | IND | NA | India | IRIS 313-8436 | JAP | T | Indonesia |
| IRIS 313-11286 | IND | NA | India | IRIS 313-8453 | IND | NA | India |
| IRIS 313-11287 | IND | NA | India | IRIS 313-8454 | IND | NA | Taiwan |
| IRIS 313-11289 | ARO | NA | India | IRIS 313-8493 | IND | T | Indonesia |
| IRIS 313-11295 | AUS | NA | India | IRIS 313-8530 | IND | T | India |
| IRIS 313-11297 | ADM | NA | India | IRIS 313-8557 | IND | T | Malaysia |
| IRIS 313-11298 | AUS | NA | India | IRIS 313-8568 | IND | T | India |
| IRIS 313-11301 | IND | NA | India | IRIS 313-8571 | IND | T | Tanzania |
| IRIS 313-11302 | IND | NA | India | IRIS 313-8585 | IND | NA | India |
| IRIS 313-11303 | IND | NA | India | IRIS 313-8586 | IND | T | Thailand |
| IRIS 313-11316 | IND | T | Indonesia | IRIS 313-8595 | IND | NA | Madagascar |
| IRIS 313-11321 | IND | T | Bangladesh | IRIS 313-8606 | IND | P | na |
| IRIS 313-11324 | AUS | T | Bangladesh | IRIS 313-8627 | JAP | I | United_States |
| IRIS 313-11338 | IND | T | Philippines | IRIS 313-8641 | AUS | P | Bangladesh |
| IRIS 313-11345 | IND | T | Philippines | IRIS 313-8658 | JAP | I | United_States |
| IRIS 313-11350 | ARO | NA | India | IRIS 313-8659 | IND | NA | Myanmar |
| IRIS 313-11351 | IND | NA | India | IRIS 313-8660 | IND | T | Sri_Lanka |
| IRIS 313-11358 | IND | NA | India | IRIS 313-8665 | JAP | I | United_States |
| IRIS 313-11370 | IND | NA | India | IRIS 313-8681 | IND | T | Guinea |
| IRIS 313-11372 | IND | NA | India | IRIS 313-8687 | JAP | T | Guinea-Bissau |
| IRIS 313-11386 | IND | T | Thailand | IRIS 313-8690 | JAP | NA | Vietnam |
| IRIS 313-11394 | IND | T | Indonesia | IRIS 313-8703 | IND | P | Bangladesh |
| IRIS 313-11395 | IND | T | Indonesia | IRIS 313-8725 | IND | T | Indonesia |
| IRIS 313-11416 | IND | NA | India | IRIS 313-8745 | JAP | NA | Haiti |
| IRIS 313-11431 | IND | I | Philippines | IRIS 313-8751 | IND | NA | Myanmar |
| IRIS 313-11435 | JAP | T | Ivory_Coast | IRIS 313-8755 | JAP | I | Japan |
| IRIS 313-11436 | JAP | T | Ivory_Coast | IRIS 313-8768 | JAP | T | Ivory_Coast |
| IRIS 313-11443 | IND | T | India | IRIS 313-8803 | JAP | I | United_States |
| IRIS 313-11453 | IND | T | India | IRIS 313-8864 | AUS | T | Bangladesh |
| IRIS 313-11460 | IND | T | India | IRIS 313-8883 | JAP | T | Malaysia |
| IRIS 313-11461 | IND | T | India | IRIS 313-8909 | IND | T | Tanzania |
| IRIS 313-11467 | IND | T | Philippines | IRIS 313-8911 | ARO | T | Thailand |
| IRIS 313-11472 | IND | T | Philippines | IRIS 313-8923 | JAP | I | United_States |
| IRIS 313-11477 | AUS | NA | India | IRIS 313-8924 | IND | T | India |
| IRIS 313-11483 | AUS | T | Bangladesh | IRIS 313-8925 | IND | T | Sri_Lanka |
| IRIS 313-11484 | AUS | T | Bangladesh | IRIS 313-8930 | IND | T | Bangladesh |
| IRIS 313-11489 | AUS | NA | India | IRIS 313-8935 | IND | NA | India |
| IRIS 313-11493 | IND | T | India | IRIS 313-8940 | IND | NA | China |
| IRIS 313-11513 | IND | NA | Ecuador | IRIS 313-8948 | IND | T | Philippines |
| IRIS 313-11515 | IND | I | na | IRIS 313-8967 | IND | NA | India |
| IRIS 313-11516 | IND | I | Philippines | IRIS 313-8982 | AUS | NA | India |
| IRIS 313-11521 | IND | T | Vietnam | IRIS 313-8985 | IND | T | Thailand |
| IRIS 313-11522 | JAP | NA | China | IRIS 313-8986 | AUS | T | India |
| IRIS 313-11528 | IND | T | Ivory_Coast | IRIS 313-8988 | IND | T | India |
| IRIS 313-11530 | IND | T | Thailand | IRIS 313-9020 | IND | T | Thailand |
| IRIS 313-11543 | IND | NA | Myanmar | IRIS 313-9023 | IND | P | India |
| IRIS 313-11546 | IND | NA | Myanmar | IRIS 313-9039 | IND | T | Sri_Lanka |
| IRIS 313-11547 | IND | NA | Myanmar | IRIS 313-9048 | JAP | T | Bhutan |
| IRIS 313-11555 | IND | T | Sierra_Leone | IRIS 313-9066 | IND | P | Bangladesh |
| IRIS 313-11567 | ARO | T | Nepal | IRIS 313-9067 | IND | T | Bangladesh |
| IRIS 313-11575 | JAP | NA | China | IRIS 313-9112 | IND | NA | Thailand |
| IRIS 313-11582 | JAP | NA | China | IRIS 313-9116 | IND | NA | Thailand |
| IRIS 313-11591 | ADM | NA | Malaysia | IRIS 313-9117 | IND | T | Indonesia |
| IRIS 313-11596 | IND | NA | India | IRIS 313-9121 | IND | T | Thailand |
| IRIS 313-11602 | AUS | NA | India | IRIS 313-9131 | IND | NA | Vietnam |
| IRIS 313-11604 | AUS | NA | India | IRIS 313-9148 | IND | P | Bangladesh |
| IRIS 313-11607 | IND | NA | India | IRIS 313-9156 | IND | I | Bangladesh |
| IRIS 313-11615 | IND | T | Guinea | IRIS 313-9160 | IND | NA | Senegal |
| IRIS 313-11617 | AUS | T | India | IRIS 313-9182 | IND | NA | Myanmar |
| IRIS 313-11618 | AUS | T | India | IRIS 313-9198 | IND | NA | Laos |
| IRIS 313-11622 | IND | NA | China | IRIS 313-9228 | JAP | P | Japan |
| IRIS 313-11624 | IND | T | Nepal | IRIS 313-9262 | IND | T | Bangladesh |
| IRIS 313-11626 | ARO | T | Nepal | IRIS 313-9294 | IND | NA | Gambia |
| IRIS 313-11630 | ARO | T | Nepal | IRIS 313-9320 | IND | T | Indonesia |
| IRIS 313-11635 | IND | T | Thailand | IRIS 313-9324 | IND | T | China |
| IRIS 313-11638 | IND | NA | India | IRIS 313-9372 | IND | NA | China |
| IRIS 313-11642 | IND | NA | India | IRIS 313-9379 | JAP | T | South_Korea |
| IRIS 313-11643 | IND | NA | India | IRIS 313-9384 | IND | T | India |
| IRIS 313-11644 | IND | NA | India | IRIS 313-9406 | IND | T | Thailand |
| IRIS 313-11645 | IND | NA | India | IRIS 313-9409 | IND | T | Malaysia |
| IRIS 313-11646 | IND | NA | India | IRIS 313-9422 | AUS | T | Bangladesh |
| IRIS 313-11647 | IND | NA | India | IRIS 313-9427 | IND | NA | India |
| IRIS 313-11648 | IND | NA | India | IRIS 313-9449 | AUS | T | Pakistan |
| IRIS 313-11651 | JAP | NA | China | IRIS 313-9464 | IND | I | Surinam |
| IRIS 313-11652 | JAP | NA | China | IRIS 313-9469 | IND | T | China |
| IRIS 313-11654 | JAP | NA | China | IRIS 313-9470 | JAP | T | Indonesia |
| IRIS 313-11655 | JAP | NA | China | IRIS 313-9472 | IND | NA | Sri_Lanka |
| IRIS 313-11656 | IND | I | Indonesia | IRIS 313-9523 | JAP | I | Japan |
| IRIS 313-11657 | IND | NA | Nigeria | IRIS 313-9570 | IND | NA | China |
| IRIS 313-11658 | JAP | T | Sierra_Leone | IRIS 313-9590 | IND | T | Indonesia |
| IRIS 313-11659 | JAP | T | Sierra_Leone | IRIS 313-9594 | IND | T | Bangladesh |
| IRIS 313-11661 | JAP | T | Bhutan | IRIS 313-9602 | IND | NA | Thailand |
| IRIS 313-11663 | IND | T | Zimbabwe | IRIS 313-9605 | IND | T | India |
| IRIS 313-11664 | IND | NA | China | IRIS 313-9626 | AUS | T | Bangladesh |
| IRIS 313-11665 | IND | NA | China | IRIS 313-9701 | JAP | I | Taiwan |
| IRIS 313-11666 | IND | NA | China | IRIS 313-9790 | JAP | NA | Uruguay |
| IRIS 313-11667 | IND | NA | China | IRIS 313-9917 | IND | T | Sri_Lanka |
| IRIS 313-11668 | IND | NA | China | IRIS 313-9922 | IND | I | South_Korea |
| IRIS 313-11669 | IND | I | China | IRIS 313-9935 | IND | NA | Guyana |
| IRIS 313-11671 | IND | T | Nepal | IRIS 313-9936 | IND | NA | Sri_Lanka |
| IRIS 313-11673 | JAP | T | Philippines | IRIS 313-9937 | JAP | NA | Italy |
| IRIS 313-11674 | IND | T | Thailand | IRIS 313-9944 | IND | NA | Solomon_Islands |
| IRIS 313-11677 | IND | T | Thailand | IRIS 313-9961 | JAP | NA | Norway |
| IRIS 313-11678 | IND | T | Thailand | IRIS 313-9963 | AUS | NA | Sri_Lanka |
| IRIS 313-11679 | IND | T | Thailand | IRIS 313-9966 | IND | P | Colombia |
| IRIS 313-11681 | IND | T | Thailand | IRIS 313-9968 | IND | T | Sri_Lanka |
| IRIS 313-11683 | IND | T | Thailand | IRIS 313-9996 | JAP | I | South_Korea |

* Status: I, improved; T, traditional; P, breeding and inbred lines (promising line); NA, unknown.

**Supplementary Table 2** Traits used in this study.

| **Phenotype** | **Recoding** | **N** | | **Mean** | | **SD** | |  |  |
| --- | --- | --- | --- | --- | --- | --- | --- | --- | --- |
| Culm Diameter | None | | 608 | | 1.62 | | 0.49 | |  |
| Culm strength | Classes {1,2,3} recoded as {1}, classes {4:9} as {2} | | 642 | | 1.39 | | 0.49 | |  |
| Flag leaf angle | None | | 639 | | 3.89 | | 1.68 | |  |
| Grain length | None | | 641 | | 8.62 | | 1.02 | |  |
| Grain width | None | | 641 | | 3.01 | | 0.39 | |  |
| Leaf length | None | | 606 | | 3.17 | | 0.68 | |  |
| Leaf senescence | Classes {2:9} recoded as {2} | | 640 | | 1.56 | | 0.49 | |  |
| Grain weight | None | | 641 | | 2.47 | | 0.49 | |  |
| Salt injury | Classes {1:7} recoded as {1}, class {9} as {2} | | 602 | | 1.46 | | 0.49 | |  |
| Time to flowering | Log transformation | | 642 | | 4.59 | | 0.23 | |  |
| Panicle threshability | Classes {1:5} recoded as {1}, classes {6:9} as {2} | | 639 | | 1.43 | | 0.49 | | |

**Supplementary Table 3** MITE family IDs from Castanera et al. (2021).

| **MITE family*** | **MITE type**** | **# TIPS** | **# Genic TIPs** | **Percentage of genic TIPs** |
| --- | --- | --- | --- | --- |
| MH63fam6_341 | Tourist-like | 3058 | 942 | 30.8 |
| MH63fam8_344 | Tourist-like | 2087 | 657 | 31.5 |
| MH63fam13_234 | MITE-adh B-like | 1166 | 475 | 40.7 |
| MH63fam14_237 | MITE-adh B-like | 1771 | 603 | 34.0 |
| MH63fam29_244 | unclassified | 1850 | 585 | 31.6 |
| MH63fam32_236 | MITE-adh B-like | 2185 | 624 | 28.6 |
| MH63fam47_235 | MITE-adh B-like | 3627 | 1312 | 36.2 |
| MH63fam50_219 | MITE-adh M-like | 1207 | 471 | 39.0 |
| MH63fam51_257 | unclassified | 696 | 238 | 34.2 |
| MH63fam72_365 | Amy/LTP-like | 971 | 354 | 36.5 |
| MH63fam73_259 | unclassified | 1003 | 292 | 29.1 |
| MH63fam106_364 | Castaway-like | 1067 | 314 | 29.4 |
| N22fam5_230 | MITE-adh I-like | 1093 | 354 | 32.4 |
| N22fam30_347 | Tourist-like | 767 | 223 | 29.1 |
| N22fam34_480 | Ditto-like | 2615 | 742 | 28.4 |
| Oryza1fam20_279 | Gaijin/Gaigin-like | 2170 | 690 | 31.8 |
| SE260500111fam211_334 | Tourist-like | 1019 | 301 | 29.5 |
| SE260500112fam219_340 | Tourist-like | 2357 | 696 | 29.5 |

** Family IDs from Castanera et al., (2021)*

*** Classification based on best BLAST hit to Oryza Repeat Database (http://rice.uga.edu/annotation_oryza.shtml)*

**Supplementary Table 4** Percentage of bootstrap samples where prediction correlation is larger with a given marker set than with SNPs only. Within Population Scenario.

| **Marker set** | **MITE/DTX > SNP** | | **RLX/RIX > SNP** | | **ALL > SNP** | |
| --- | --- | --- | --- | --- | --- | --- |
|  | **BayesC** | **RKHS** | **BayesC** | **RKHS** | **BayesC** | **RKHS** |
| Culm Diameter | 0.90 | 0.92 | 0.63 | 0.83 | 0.99 | 0.99 |
| Culm strength | 0.28 | 0.12 | 0 | 0.08 | 0.22 | 0.12 |
| Flag leaf angle | 0.05 | 0.07 | 0 | 0.09 | 0.06 | 0.09 |
| Grain length | 0.82 | 0.20 | 0 | 0.10 | 0.90 | 0.57 |
| Grain width | 0.36 | 0.05 | 0 | 0.05 | 0.64 | 0.36 |
| Leaf length | 0.67 | 0.82 | 0 | 0.69 | 0.50 | 0.85 |
| Leaf senescence | 0.52 | 0.52 | 0 | 0.79 | 0.64 | 0.69 |
| Grain weight | 0.70 | 0.81 | 0 | 0.55 | 0.76 | 0.59 |
| Salt injury | 0.53 | 0.32 | 0 | 0.34 | 0.34 | 0.41 |
| Time to flowering | 0.78 | 0.89 | 0 | 0.48 | 0.59 | 0.59 |
| Pan. threshability | 0.24 | 0.18 | 0 | 0.29 | 0.28 | 0.21 |

*ALL: All marker model*

**Supplementary Table 5** Percentage of bootstrap samples where prediction correlation is larger with a given marker set than with SNPs only. Across Population Scenario.

| **Marker** | **MITE/DTX > SNP** | | **RLX/RIX > SNP** | | **ALL > SNP** | |
| --- | --- | --- | --- | --- | --- | --- |
|  | **BayesC** | **RKHS** | **BayesC** | **RKHS** | **BayesC** | **RKHS** |
| Culm Diameter | 0.83 | 0.92 | 0.18 | 0.37 | 0.95 | 0.81 |
| Culm strength | 0.79 | 0.82 | 0.83 | 0.89 | 0.81 | 0.81 |
| Flag leaf angle | 0.39 | 0.67 | 0.83 | 0.92 | 0.14 | 0.67 |
| Grain length | 0.02 | 0.67 | 0.02 | 0.22 | 0.68 | 0.84 |
| Grain width | 0.93 | 0.99 | 0.04 | 0.99 | 0.27 | 0.99 |
| Leaf length | 0.62 | 0.62 | 0.70 | 0.91 | 0.96 | 0.89 |
| Leaf senescence | 0.98 | 0.97 | 0.97 | 0.98 | 0.99 | 0.98 |
| Grain weight | 0.74 | 0.58 | 0.30 | 0.37 | 0.95 | 0.83 |
| Salt injury | 0.00 | 0.01 | 0.52 | 0.13 | 0.22 | 0.04 |
| Time to flowering | 0.19 | 0.73 | 0.09 | 0.41 | 0.83 | 0.94 |
| Pan. threshability | 0.06 | 0.15 | 0.02 | 0.01 | 0.23 | 0.18 |

*ALL: All marker model*

**Supplementary Table 6** Correlation between observed and predicted phenotypes under a linear and threshold model.

| **Model** | **Linear** | | | **Threshold** | | |
| --- | --- | --- | --- | --- | --- | --- |
|  | **SNPs** | **MITE /DTX** | **RLX/ RIX** | **SNPs** | **MITE/ DTX** | **RLX/ RIX** |
| Culm Diameter | -0.07 | 0.26* | 0.09 | 0.13 | 0.26* | 0.06 |
| Culm Strength | 0.05 | 0.20* | 0.18 | -0.04 | 0.11 | 0.15 |
| Flag Leaf Angle | 0.00 | 0.11 | 0.14 | 0.10 | 0.13 | 0.26* |

** Best strategy*

**Supplementary Table 7** Root Mean Squared Error Value (RMSE): Within Population Scenario

| **Marker** | **SNPs** | | **MITE/DTX** | | **RLX/RIX** | | **ALL** | |
| --- | --- | --- | --- | --- | --- | --- | --- | --- |
| **Method** | **BayesC** | **RKHS** | **BayesC** | **RKHS** | **BayesC** | **RKHS** | **BayesC** | **RKHS** |
| Culm Diameter | 1.05 | 1.04 | 1.03 | 1.01* | 1.06 | 1.04 | 1.03 | 1.02 |
| Culm strength | 0.99* | 1.00 | 1.01 | 1.04 | 1.04 | 1.06 | 1.01 | 1.04 |
| Flag leaf angle | 1.04* | 1.05 | 1.14 | 1.14 | 1.14 | 1.13 | 1.06 | 1.08 |
| Grain length | 0.79 | 0.82 | 0.76* | 0.85 | 0.87 | 0.86 | 0.77 | 0.82 |
| Grain width | 0.43* | 0.51 | 0.49 | 0.63 | 0.60 | 0.63 | 0.43* | 0.53 |
| Leaf length | 0.97 | 0.98 | 0.97 | 0.97 | 1.00 | 0.97 | 0.97 | 0.96* |
| Leaf senescence | 0.89 | 0.90 | 0.89 | 0.90 | 0.89 | 0.86* | 0.88 | 0.88 |
| Grain weight | 0.82 | 0.81 | 0.79 | 0.78* | 0.81 | 0.79 | 0.81 | 0.80 |
| Salt injury | 0.95* | 0.96 | 0.96 | 0.96 | 0.97 | 0.96 | 0.96 | 0.95* |
| Time to flowering | 0.61* | 0.61* | 0.69 | 0.65 | 0.66 | 0.68 | 0.61* | 0.62 |
| Pan. threshability | 0.96 | 0.95* | 0.98 | 0.97 | 0.97 | 0.95* | 0.97 | 0.96 |

*Asterisk * indicates the lowest value*

*ALL: All marker model*

**Supplementary Table 8** Root Mean Squared Error Value (RMSE): Across Population Scenario

| **Marker** | **SNPs** | | **MITE/DTX** | | **RLX/RIX** | | **ALL** | |
| --- | --- | --- | --- | --- | --- | --- | --- | --- |
| **Method** | **BayesC** | **RKHS** | **BayesC** | **RKHS** | **BayesC** | **RKHS** | **BayesC** | **RKHS** |
| Culm Diameter | 0.96 | 0.97 | 0.94* | 0.95 | 0.98 | 0.98 | 0.95 | 0.96 |
| Culm strength | 1.00 | 1.01 | 0.98* | 0.99 | 0.98* | 0.98* | 0.98 | 1.01 |
| Flag leaf angle | 0.96 | 0.98 | 0.95 | 0.96 | 0.92* | 0.93 | 0.96 | 0.97 |
| Grain length | 1.17* | 1.41 | 1.40 | 1.39 | 1.42 | 1.46 | 1.17* | 1.38 |
| Grain width | 1.24 | 1.29 | 0.99* | 1.14 | 1.32 | 1.18 | 1.28 | 1.23 |
| Leaf length | 0.89 | 0.89 | 0.88 | 0.89 | 0.87 | 0.84* | 0.86 | 0.86 |
| Leaf senescence | 1.02 | 0.99 | 0.88* | 0.88* | 0.91 | 0.88* | 0.89 | 0.89 |
| Grain weight | 1.12 | 1.23 | 1.10* | 1.11 | 1.17 | 1.14 | 1.11 | 1.11 |
| Salt injury | 0.96 | 0.97 | 1.00 | 0.99 | 0.95* | 0.97 | 0.96 | 0.98 |
| Time to flowering | 0.79 | 0.78 | 0.76 | 0.77 | 0.79 | 0.76 | 0.78 | 0.75* |
| Pan. threshability | 0.96* | 0.98 | 0.99 | 0.99 | 0.99 | 1.02 | 0.97 | 0.99 |

*Asterisk * indicates the lowest value*

*ALL: All marker model*


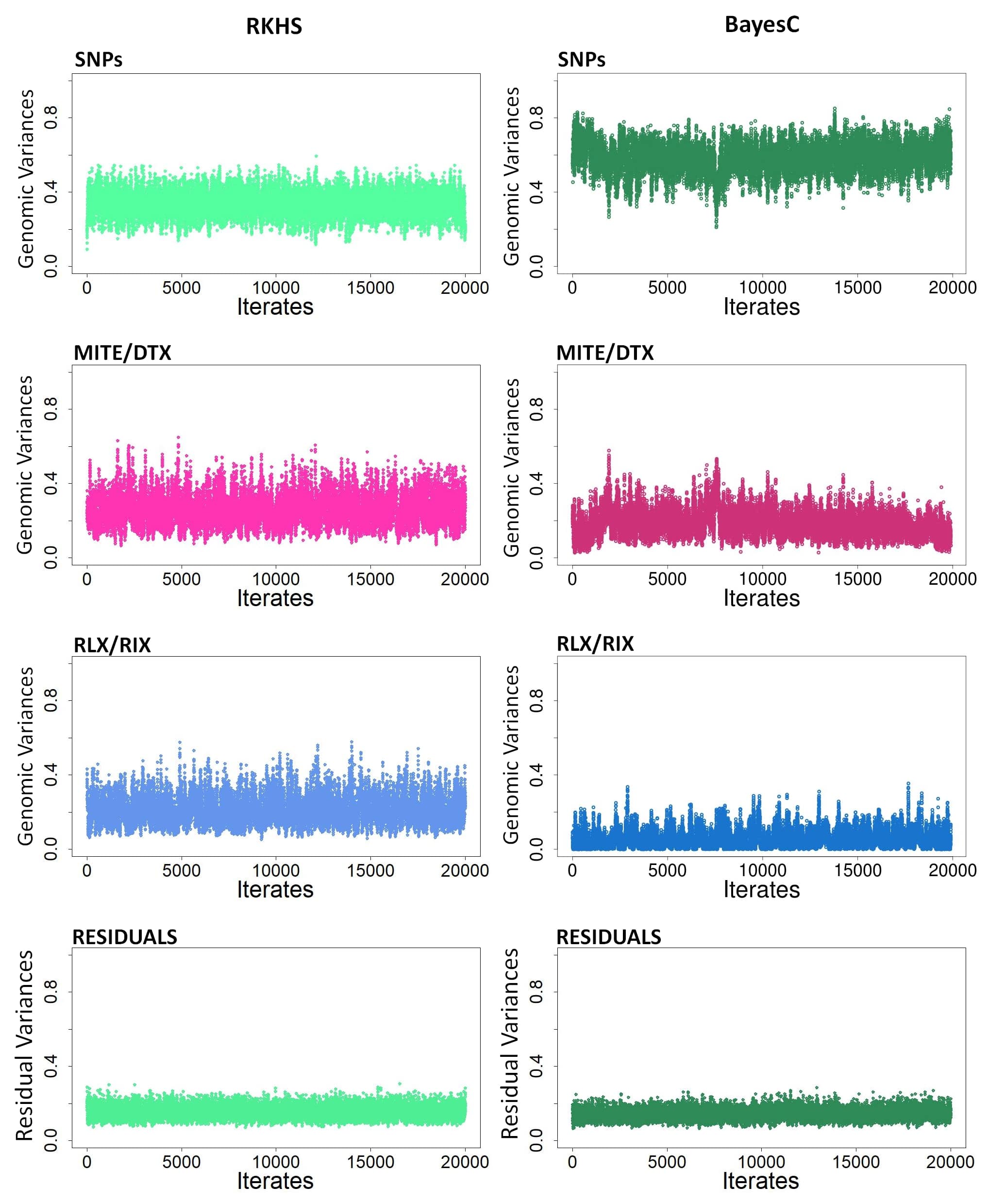


**Supplementary Fig. 1** Plot of variances across iterations to show convergence. Results correspond to “Grain width” under models 1a and 2a. Variances with Bayes C were computed as in <https://github.com/gdlc/BGLR-R/blob/master/inst/md/heritability.md>.


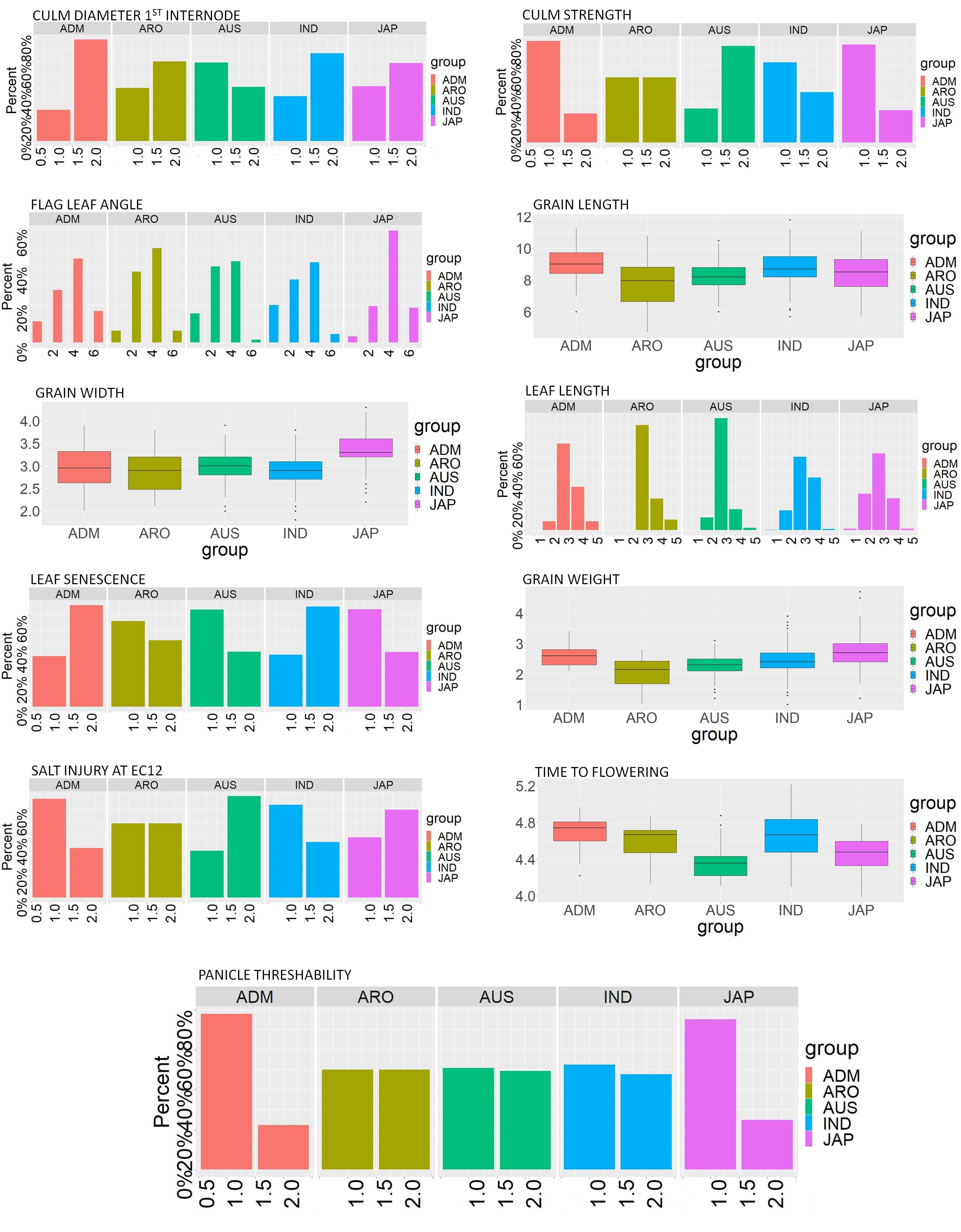


**Supplementary Fig. 2** Raw phenotypic distributions by populations, each shown in a different color.


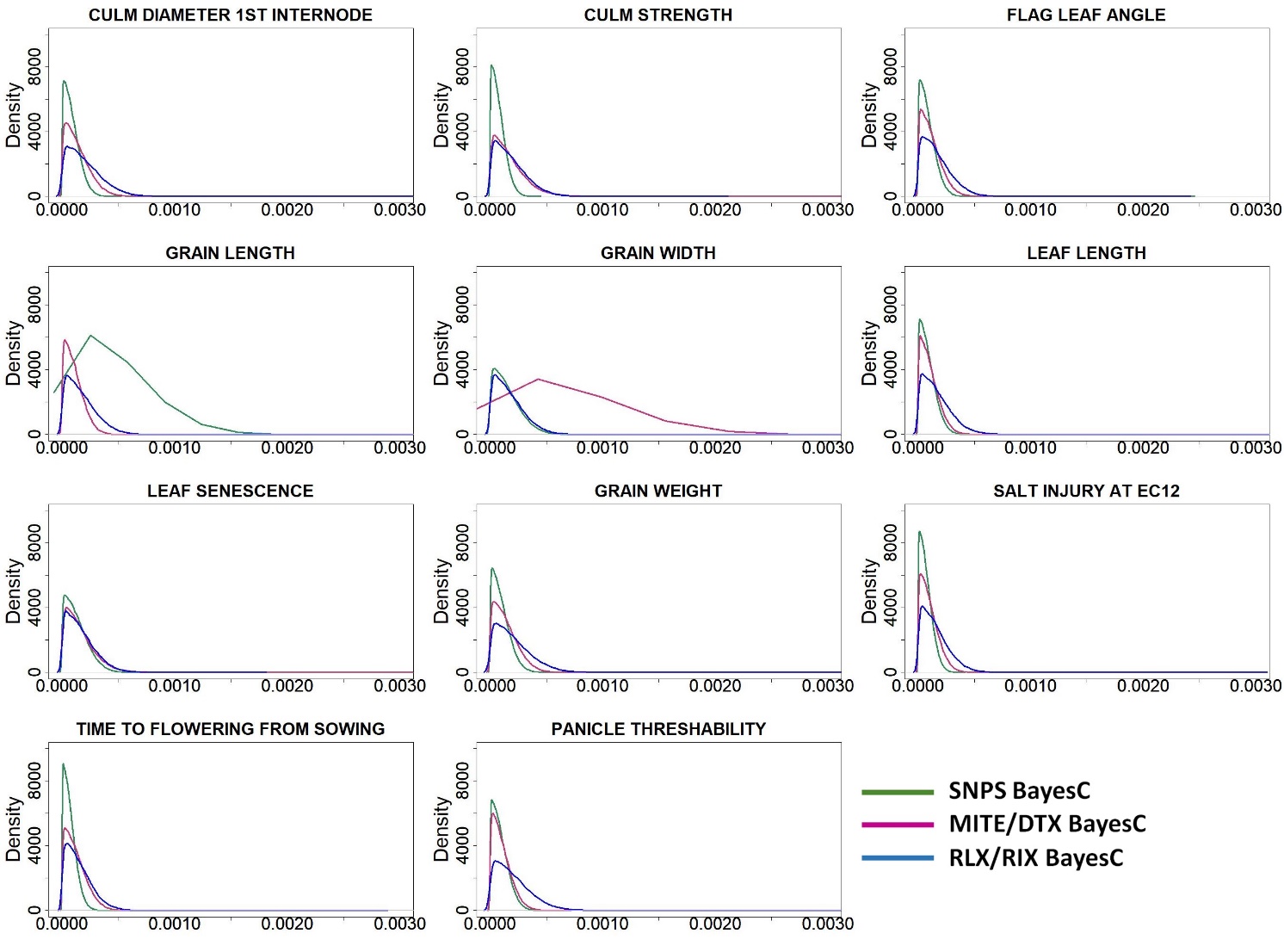


**Supplementary Fig. 3** Distributions of estimated marker effects from Bayes C using model 2a in the across population prediction scenario.


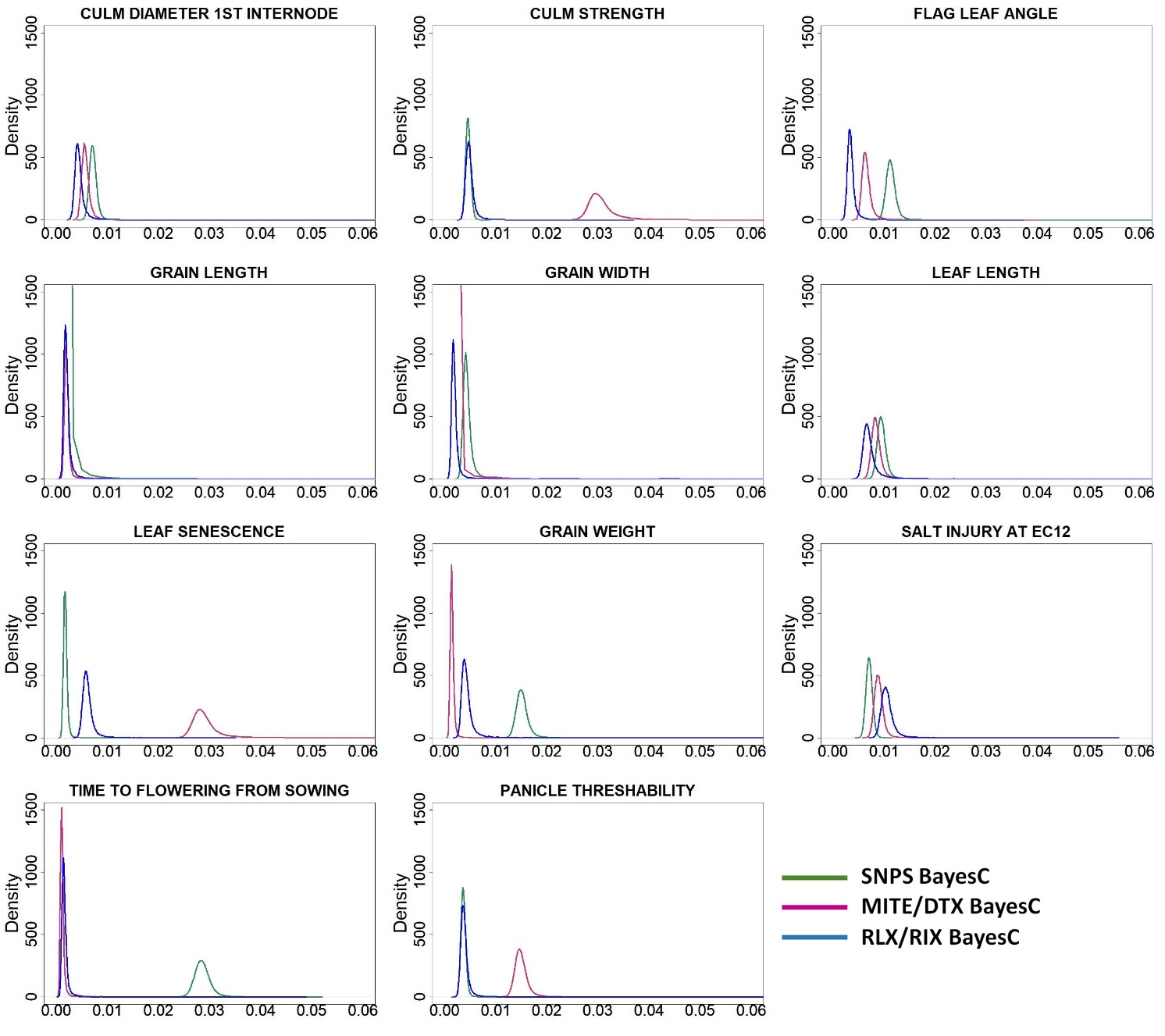


**Supplementary Fig. 4** Distributions of marker probabilities entering the model (d) in Bayes C using model 2a in the across population prediction scenario.
